# RhoA deficiency in chondrocyte inhibits cartilage fibrosis and ameliorates osteoarthritis progression via SOX4/MMP2 axis

**DOI:** 10.1016/j.jot.2026.101127

**Published:** 2026-05-14

**Authors:** Yizhou Xu, Shuyi Xu, Jiayi Li, Shicheng Wang, Jie Liang, Jiaqi Wang, Xianghai Wang, Gang Deng, Lixin Zhu, Jiasong Guo

**Affiliations:** a Department of Spinal Surgery, Orthopedic Medical Center, Zhujiang Hospital, Southern Medical University, Guangzhou, 510280, China; b Department of Histology and Embryology, School of Basic Medical Sciences, Southern Medical University, Guangzhou, 510515, China; c Department of Sport Medicine, Ganzhou Hospital-Nanfang Hospital, Southern Medical University, Ganzhou, 341000, China; d Department of Spinal Surgery, Guangzhou Hospital of Integrated Traditional and Western Medicine, Guangzhou, 510800, China

**Keywords:** Actin cytoskeleton, Cartilage, Chondrocytes, Fibrosis, Osteoarthritis, RhoA

## Abstract

**Background:**

Cartilage fibrosis plays a critical role in the onset and progression of osteoarthritis (OA), and although RhoA is a well-known small GTPase that regulates cytoskeletal reorganization, its role in OA progression remains inadequately explored.

**Methods:**

In this study, we first screened public scRNA-seq datasets for genes enriched in fibrocartilage chondrocytes and found that *RHOA* is significantly upregulated in fibrocartilage chondrocytes within OA cartilage obtained from patients undergoing total knee arthroplasty. Then, we induced post-traumatic OA in 8-week-old male C57BL/6J mice by destabilization of the medial meniscus and generated chondrocyte-specific *Rhoa* deletion using *Col2a1*-CreERT-*Rhoa*-flox/flox mice. After that, cartilage damage was graded by Safranin-O/Fast Green, Toluidine blue, and Micro-CT, and molecular changes were validated by Immunofluorescence and Western blot, leading to the identification—by integrated single-cell and bulk RNA-seq—of a β-catenin/SOX4/MMP2 axis downstream of RhoA. Finally, we injected AAV-*Sox4* or AAV-*Mmp2* intra-articularly to rescue the loss of RhoA function.

**Results:**

The results showed that conditional knockout of *Rhoa* in chondrocytes resulted in a marked reduction in cartilage fibrosis and a concurrent decrease in extracellular matrix degradation in OA mice. Mechanistically, the integrated single-cell and bulk tissue transcriptomic analyses indicated that RhoA promotes the chondrocyte transition to a fibrotic phenotype through the novel β-catenin/SOX4/MMP2 pathway, while notably, intra-articular delivery of adeno-associated viral vectors overexpressing *Sox4* or *Mmp2* reversed the phenotypes of *Rhoa*-deficient mice.

**Conclusion:**

These findings position *RHOA* as a central regulator of chondrocyte fibrosis and as a promising therapeutic target for OA treatment.

**The translational potential of this article:**

These findings highlight that RhoA may represent a therapeutic target for ameliorating cartilage fibrosis and the OA process via targeted gene intervention.

## Graphical abstract

**Figure:**
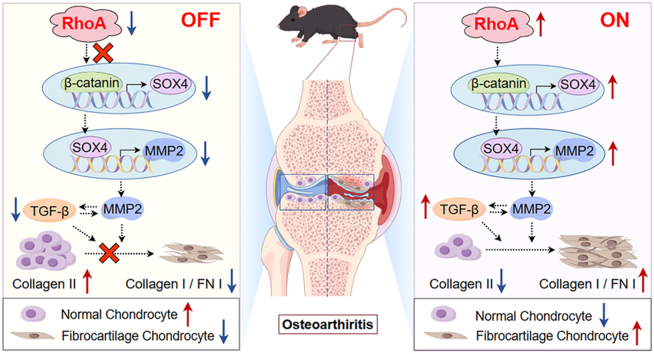

## 1 Introduction

Osteoarthritis (OA) affects over 600 million people globally. However, no treatment capable of achieving a complete cure is currently available [1,2]. The primary challenge lies in the undefined molecular determinants that trigger and sustain cartilage destruction. Emerging evidence highlights cartilage fibrosis as a key pathogenic event in OA progression [[3], [4], [5]]. Under abnormal mechanical and inflammatory stimuli, resident hyaline-cartilage chondrocytes undergo phenotypic transdifferentiation, characterized by increased secretion of Collagen I and Fibronectin 1, alongside reduced production of Collagen II [6]. This fibrotic phenotype not only impairs the compressive strength of cartilage but also accelerates extracellular matrix degradation through paracrine signaling, creating a self-amplifying loop of inflammation and mechanical dysfunction [7]. While TGF-β, Wnt/β-catenin, and integrin–FAK signaling have been implicated, therapeutic targeting of these pathways has not yielded clinical success, suggesting that crucial regulatory mechanisms remain unidentified [[8], [9], [10]]. Therefore, identifying and functionally validating a master molecular driver of chondrocyte fibrosis could offer a breakthrough for halting cartilage degeneration in OA [11].

Based on the reanalysis of a published single-cell sequencing data from clinical OA samples [12], our preliminary study revealed that the "Regulation of actin cytoskeleton" pathway was significantly enriched in the fibrocartilage chondrocytes. This finding suggests that actin cytoskeleton remodeling is closely linked to the acquisition of the fibrotic phenotype, since the actin cytoskeleton remodeling during OA progression leads to the phenotypic shift of chondrocytes from round to spindle-shaped forms [13,14]. Notably, within this enriched pathway, *RHOA* emerged as the most highly enriched key regulatory factor. As a core member of the Rho-GTPase family, RhoA is recognized as a key regulator of the actin cytoskeleton, often upregulated following tissue injury. Its classical function involves promoting actin stress fiber formation via the RhoA/ROCK cascade [15]. Recent studies also suggest that RhoA’ expression or activation in different cells might play different roles in some diseases progression, and in some cell types, RhoA can influence transcriptional regulation through various non-classical pathways [16]. However, to date, only 5 studies have implicated RhoA is involved in endothelial cells ferroptosis, synovial macrophage activation, and drug treatment in OA tissues [[17], [18], [19], [20], [21]], but none of them explored the role of chondrocytic RhoA in OA, particularly in the cartilage fibrosis and the fate of chondrocytes.

In this study, database analysis revealed significant upregulation of the RhoA-associated actin cytoskeleton signaling pathway in the OA fibrocartilage chondrocytes population. This finding was further confirmed by Western blotting and immunostaining in clinical OA specimens and animal models. To elucidate the role and underlying mechanism of chondrocytic RhoA in OA progression, a mouse model with chondrocyte-specific *Rhoa* knockout was developed. The collected data demonstrated that *Rhoa* knockout in chondrocytes significantly alleviated cartilage fibrosis and deferred OA progression.

Notably, RhoA was found to regulate fibrocartilage chondrocytes not via the conventional RhoA/ROCK signaling pathway as previously assumed, but through a novel β-Catenin/SOX4/MMP2 signaling axis. This is the first study to report that OA induces upregulation of RhoA expression in chondrocytes, where it plays a pivotal role in driving fibrosis via unconventional signaling pathway. These findings provide a new insight for understanding the pathological mechanisms of OA and RhoA's bio-functions, while also implicate targeting RhoA/β-Catenin/SOX4/MMP2 axis is a potential therapeutic strategy for OA treatment.

## 2 Materials and methods

### 2.1 Collection and processing of clinical samples

Human articular cartilage was obtained from two sources. OA cartilage (n = 4) was harvested from patients undergoing total knee arthroplasty at Ganzhou Hospital–Nanfang Hospital. The OA cohort had a mean age of 65 ± 3 years (2 females and 2 males), all classified as Kellgren–Lawrence Grade IV, without major systemic comorbidities. Normal control cartilage (n = 4) was obtained from age-matched patients (mean age 65 ± 3 years; 2 females and 2 males) who required lower-limb amputation after severe trauma but had macroscopically intact knee joints and no pre-existing osteoarthritis or chronic comorbidities. All participants provided written informed consent, and the study protocol was approved by the Ethics Committee of Ganzhou Hospital–Nanfang Hospital (approval no. TY-ZKY2024-141-01). Each cartilage sample was processed immediately after surgical removal as follows. (i) Snap-frozen in liquid nitrogen and stored at −80 °C for subsequent protein extraction and Western blotting. (ii) Fixed in 10 % neutral-buffered formalin for 72 h, decalcified with 10 % EDTA, and embedded in paraffin for histological sectioning. (iii) Used for ex-vivo culture. Full-thickness cartilage slices were prepared with a scalpel, trimmed into ∼1 cm^3^ explants under sterile conditions, and transferred to complete medium supplemented with 10 % fetal bovine serum for the experiments described below.

### 2.2 Primary cell culture and in vitro fibrosis induction model

Primary chondrocytes were isolated from knee joints of 3-day-old C57BL/6 neonatal mice by enzymatic digestion with 0.2% type II collagenase (2 h, 37 °C). Cells were cultured in DMEM/F-12 supplemented with 10% fetal bovine serum (FBS) and 1% penicillin –streptomycin, and used within two passages (passage ≤2) [21]. For adult mouse chondrocyte isolation, knee cartilage was harvested and digested according to published protocols [22].

Chondrocytes were stimulated with a pro-inflammatory cocktail containing 5 ng/ml IL-1β and 25 ng/ml TNF-α for 24 h to induce fibrosis, and assessed by Collagen I and Fibronectin 1 expression [5].

### 2.3 Establishment of OA animal model

Sixteen healthy adult male C57BL/6J wild-type mice (25–30 g) were randomized into two groups (n = 8 per group): (i) a sham-operated control group and (ii) a DMM (destabilization of the medial meniscus) group. After intraperitoneal tribromoethanol anesthesia (180 mg kg^−1^), the right knee was shaved and disinfected. A medial parapatellar incision was made; the joint capsule was opened and the patella luxated laterally. In the DMM group, the medial meniscotibial ligament was transected to destabilize the medial meniscus; in the sham group the joint was exposed without ligament transection. The incision was closed in layers. Carprofen (5 mg kg^−1^) was administered once for post-operative analgesia, and mice were monitored until fully recovered. Eight weeks after surgery, mice were euthanized and the right knee joints harvested. Cartilage from half of the joints was snap-frozen for Western blot analysis; the remaining joints were fixed in 4 % paraformaldehyde (72 h), decalcified in 10 % EDTA (30 d), and embedded in paraffin for 5-μm sections. All experimental procedures were conducted in compliance with protocols approved by the Animal Care and Use Committee of Southern Medical University, ensuring adherence to ethical standards.

### 2.4 Genotyping

Genomic DNA was extracted from 2 to 3 mm tail clips (overnight digestion at 55 °C in modified Gitschier buffer, proteinase K 1 mg mL^−1^, heat-inactivated 90 °C 10 min), and 1–2 μL lysate was used for PCR with gene-specific primers; amplicons were resolved on 1.5 % agarose gels. Genotyping was performed as previously described [23]. Experimental mice: (i) *Col2a1*^−CreERT^-*Rhoa*^-flox/flox^ mice (chondrocyte-specific *Rhoa* conditional knockout, cKO) and (ii) *Col2a1*^−CreERT^ littermates (Cre-only controls).

### 2.5 Generation and validation of chondrocyte-specific *Rhoa* conditional knockout mice

To ensure comparable cartilage development and minimize pre-existing differences, we used a tamoxifen-inducible Cre-loxP system. Eight-week-old *Col2a1*^−CreERT^-*Rhoa*^-flox/flox^ (cKO) mice and *Col2a1*^−CreERT^ (Cre) received intraperitoneal tamoxifen (100 mg kg^−1^ day^−1^) for five consecutive days. Cre-positive mice were selected as controls to ensure both groups received identical tamoxifen treatment and to control for any potential non-specific effects of the Cre transgene itself, thereby isolating RhoA deletion as the sole experimental variable [24,25]. Ten days after the last injection, a subset of mice was euthanized; knee-joint cartilage was harvested for Western blot and immunofluorescence to confirm *Rhoa* deletion. Only mice with verified chondrocyte-specific knockout (cKO) or intact RhoA expression (Cre) were subsequently randomized into four experimental groups: (i) Sham-Cre (sham-operated Cre control), (ii) Sham-cKO (sham-operated RhoA knockout), (iii) DMM-Cre (DMM surgery with Cre control), and (iv) DMM-cKO (DMM surgery with RhoA knockout). Joints were collected eight weeks post-surgery for downstream analyses. *Col2a1*^−CreERT^ and *Rhoa*^-flox/flox^ strains were obtained from Sangon Biotech (Suzhou, China).

### 2.6 Intra-articular AAV2 administration

All recombinant AAV2 vectors were produced and packaged by Ma Jin Biotech (Guangzhou, China). The vectors comprised (i) AAV2-Null (empty capsid), (ii) AAV2-*Sox4* carrying full-length murine *Sox4* cDNA, and (iii) AAV2-*Mmp2* carrying full-length murine *Mmp2* cDNA. Three days after DMM surgery, mice received a single intra-articular injection of 1 × 10^11^ vector genomes in 10 μL sterile PBS through the patellar tendon approach using a 30 G insulin syringe. Eight weeks later, joints were harvested. Western blot confirmed robust SOX4 and MMP2 over-expression in the respective AAV groups, validating successful in-vivo transduction.

### 2.7 Micro-CT analysis

Knee joints were fixed in 4 % paraformaldehyde for 72 h and scanned by micro-computed tomography (ZKKS-MicroCT 4.1, China) following established protocols. The following trabecular parameters were quantified: osteophyte number, trabecular separation (Tb. Sp, mm), trabecular thickness (Tb. Th, mm) and trabecular number per millimetre (Tb. N).

### 2.8 Histological staining

Knee joints were decalcified in 10 % (w/v) EDTA for 30 days, dehydrated through a graded ethanol series, paraffin-embedded, and then sectioned to a thickness of 5 μm. For immunofluorescence, sections were permeabilized with 0.3 % Triton X-100 for 15 min, blocked with 5 % fish gelatin for 1 h, and incubated overnight at 4 °C with primary antibodies. After three PBS washes, sections were incubated with Alexa Fluor 488- or 568-conjugated secondary antibodies for 2 h at room temperature. Antibody details are listed in Supplementary Table 1.

### 2.9 Western blotting

Cells/tissues were lysed in RIPA plus inhibitors, quantified(BCA), and immunoblotted as previously described [26]; antibody list is given in Supplementary Table 1.

### 2.10 Multimodal integration analysis

(i) Transcriptome profiling: a. Bulk RNA-seq. Articular cartilage from the tibial plateau was snap-frozen in liquid nitrogen, pulverised under cryo-conditions, and total RNA extracted with TRIzol reagent. After DNase I digestion, only samples with RNA integrity (RIN) ≥ 8 were retained. Libraries were prepared with the Illumina TruSeq Stranded mRNA kit, following the manufacturer's protocol, and sequenced on an Illumina NovaSeq 6000 (150 bp paired-end, ≥20 million reads per sample). b. Single-cell RNA-seq. Cartilage was minced and enzymatically dissociated (0.2 % type II collagenase + 0.05 % trypsin-EDTA, 37 °C, 45 min). The resulting cell suspension was filtered (70 μm), treated with ACK lysis buffer, and assessed for viability (≥92 %). Approximately 8000–10 000 cells per channel were loaded onto a 10x Genomics Chromium Controller (v3.1 chemistry). cDNA synthesis, library construction, and index PCR yielded libraries with a mean insert size of 350 bp, which were sequenced on the same NovaSeq instrument (150 bp paired-end, ≥20 000 reads per cell). c. Data processing. Raw reads were quality-filtered (FastQC, fastp) and mapped to the mm10 reference genome (STAR v2.7.9a). Gene-level quantification was performed with featureCounts. Bulk differential expression (DE) analyses used DESeq2 (|log_2_FC| > 1, FDR <0.05); scRNA-seq analyses used Seurat v4.1 (|log_2_FC| > 0.25, adjusted p < 0.05).
(ii) Multi-omics cross-validation and functional inference. a. Cell-type definition. Unsupervised graph-based clustering (Seurat, resolution = 0.8) followed by annotation with established chondrocyte markers was used to define the major cartilage-resident subpopulations. Based on differential expression analysis and published literature [12,[27], [28], [29]], six distinct chondrocyte subsets were identified with the following specific marker genes: Homeostatic chondrocytes (*Kmt2e*, *Clk1*, *Ier5*, *Wwp2*, *Ppp1r10*); Proliferative chondrocytes (*Ell22*, *Pdlim4*, *Odc11*, *Txnrd11*, *Ngf1*); Regulatory chondrocytes (*H2afz*, *Ifitm31*, *Glrx*, *Cks2*, *Sod2*); Fibrocartilage chondrocytes (*Col1a1*, *Col1a2*, *Mmp2*, *Zfp36l1*, *Olfml3*); Angiogenesis chondrocytes (*Ecrg42*, *Smoc21*, *Mmp31*); and Effector chondrocytes (*Ugp2*, *Slc29a1*, *Tspan2*, *Tbcb*, *Acly*). b. Differential gene sets. • Bulk: DEG_bulk (|log_2_FC| > 1, FDR <0.05). • Single-cell: DEG_sc (Fibrotic-Chon vs. all others, Wilcoxon rank-sum, |log_2_FC| > 0.25, adjusted p < 0.05). c. Overlap and network analysis. Overlapping genes (DEG_bulk ∩ DEG_sc) were obtained with the VennDiagram package. GO (biological process, molecular function) and KEGG pathway enrichment (clusterProfiler v4.2; org.Mm.eg.db v3.15; KEGG release 2023-03) were performed at FDR <0.05. A protein–protein interaction network was built in Cytoscape 3.9 using STRING, and hub genes were identified with CytoHubba (intersection of MCC and Degree algorithms). d. Visualisation. Volcano plots, heat maps, and network diagrams were generated in R and Cytoscape. (Sequencing services were provided by Personal Biotechnology, Shanghai, China).

### 2.11 Statistical analyses

Data are presented as mean ± standard error of the mean (SEM). Statistical analyses were performed with Prism Version 8.0.2 (GraphPad). Group comparisons were conducted with unpaired t-tests, one-way or two-way ANOVA, as appropriate. When ANOVA indicated significance, Tukey's post-hoc test was applied. Data are presented as mean ± SD from at least three independent experiments or biological replicates (n ≥ 3). A two-tailed P < 0.05 was considered statistically significant.

## 3 Results

### 3.1 The single-cell sequencing bioinformatics analysis revealed that *RHOA* is significantly upregulated in the fibrocartilage chondrocytes of OA patients

**Fig. 1:**
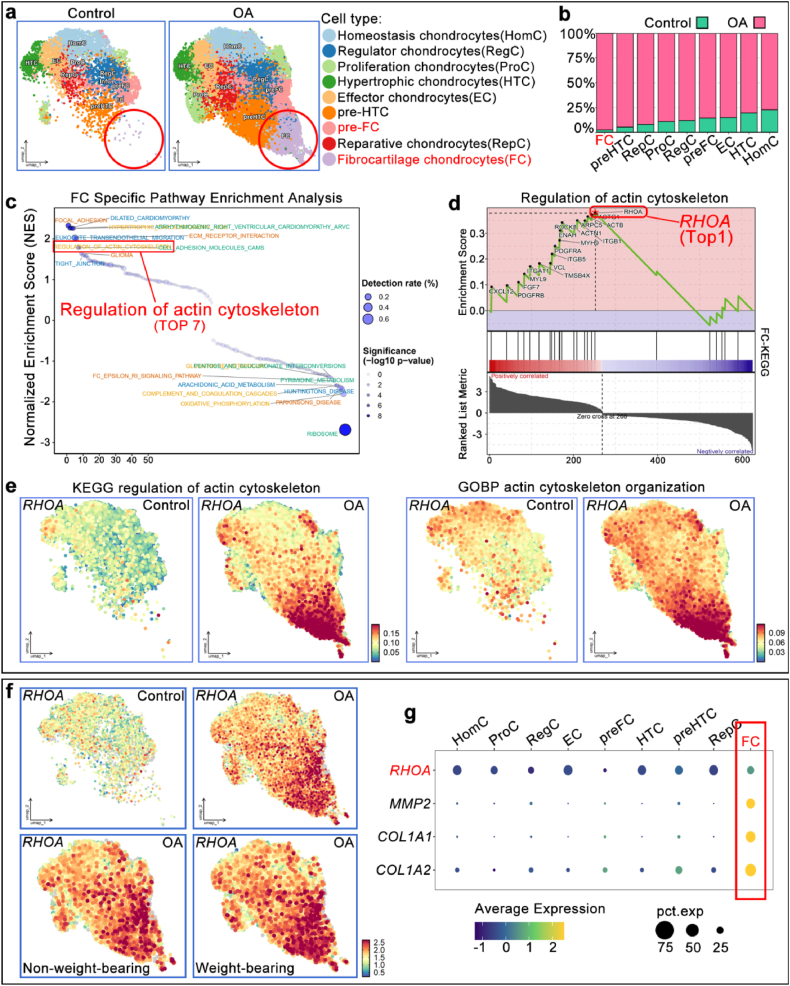
**Analysis of potential regulatory molecules involved in cartilage fibrosis based on the single-cell sequencing database of cartilage tissue of OA patients.** **a, b.** Differences in the quantity and distribution of cartilage cell subpopulations between the Control and OA groups. **c.** GSEA enrichment analysis of significantly altered characteristic KEGG signaling pathways in the fibrocartilage chondrocytes. **d.**GSEA enrichment analysis of the main molecular alterations in the fibrocartilage chondrocytes cytoskeleton regulatory pathway (hsa04810: Regulation of actin cytoskeleton). **e.** The AddModuleScore algorithm assigns scores to the KEGG and GO pathways related to the cytoskeleton, and the mapping graph shows the distribution and expression levels of pathway information in each chondrocyte subpopulation. **f.** Expression and distribution differences of *RHOA* in each cartilage cell subpopulation between the Control and OA groups, and analysis of *RHOA* expression in the non-weight-bearing and weight-bearing areas of the femoral medial condyle. **g.** Bubble chart shows *RHOA* expression intensity in each cell subpopulation.

To elucidate the pathological molecular mechanisms underlying OA cartilage fibrosis and identify potential therapeutic targets, the single-cell sequencing database of cartilage tissues from patients with OA (GSE255460 [12]) was first analyzed, categorizing the cartilage cells into subgroups. The results revealed that, compared to the control group, fibrocartilage chondrocytes represented the most significantly increased subgroup in the OA group (Fig. 1a and b). Gene enrichment analysis of the fibrocartilage chondrocytic KEGG pathways indicated that the actin cytoskeleton regulation pathway was positively correlated and ranked among the top 7 (Fig. 1c). Further GSEA analysis of the enriched genes within the cytoskeleton regulation pathways of the fibrocartilage chondrocytes clusters revealed that *RHOA* gene enrichment score ranked first (Fig. 1d). Notably, *RHOA* was highly expressed in the cytoskeleton regulation KEGG and GO functional enrichment of OA cartilage cells and was predominantly found in the fibrocartilage chondrocytes, compared to the Control group (Fig. 1e). Subsequently, a detailed analysis of *RHOA*'s expression and distribution in each major subgroup of OA cartilage cells was performed. The results showed that *RHOA* was most highly expressed in the fibrocartilage chondrocytes of OA, with higher expression in the weight-bearing area of the femoral medial condyle compared to the non-weight-bearing area (Fig. 1f and g).

### 3.2 RhoA is significantly upregulated in the joint tissues of OA patients and in the chondrocytes of the mouse OA model

**Fig. 2:**
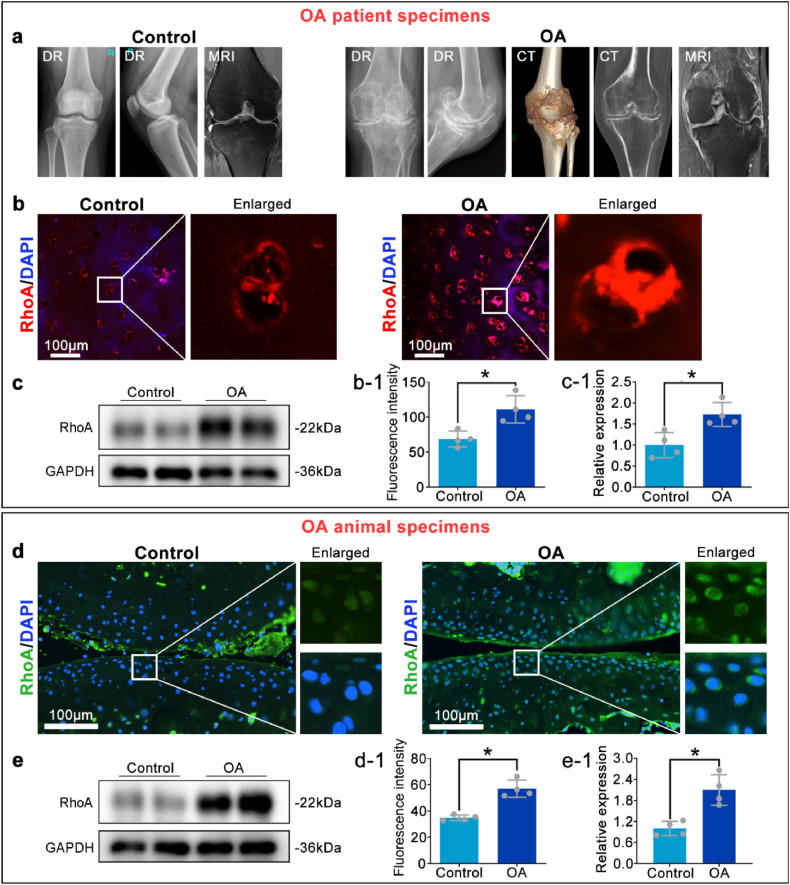
RhoA may regulate chondrocyte biological functions, influencing OA progression. **a.** Imaging of the patient's knee joint to assess OA severity. **b, c.** Immunofluorescence staining and Western blotting show the RhoA expression in the cartilages of control and OA patient. **d, e.** RhoA immunofluorescence staining and Western blotting in control and OA mouse cartilage tissue. ∗P < 0.05.

To validate the bioinformatics analysis results, chondrocyte tissues were collected from patients with OA during clinical surgeries (Fig. 2a), and a mouse OA model was established according to published protocols [26]. Immunofluorescence staining revealed a significant increase in RhoA fluorescence intensity in the chondrocytes of both OA patients and OA mice (Fig. 2b–d). Western blots also showed a significant elevation in the total protein expression level of RhoA in OA patients and OA mice (Fig. 2c–e).

### 3.3 Construction of a mouse model with conditional knockout of *Rhoa* in chondrocytes

**Fig. 3:**
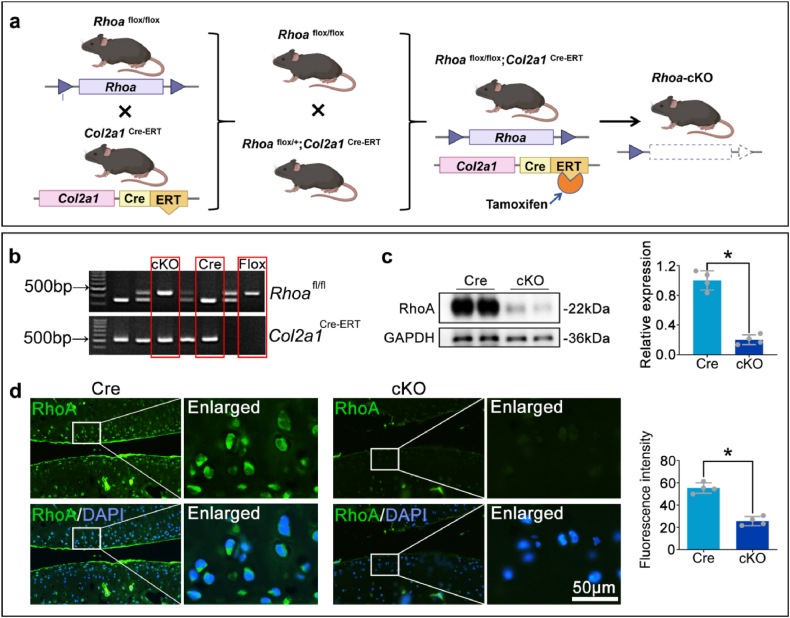
Construction and identification of chondrocytic *Rhoa* conditional knockout mice. **a.** Schematic of the transgenic mouse breeding pattern. **b.** Southern blots show the genotyping of the transgenic mice. **c, d.** Western blot and immunofluorescence staining illustrate the RhoA expression in cartilage tissue. ∗P < 0.05.

Previous studies on RhoA and OA predominantly relied on drug interventions, the observed effects may result from a combination of direct and indirect actions, since drugs inevitably affect multiple cell types simultaneously. To accurately explore the potential role of chondrocytic RhoA upregulation in OA progression, *Col2a1*^Cre−ERT^ mice were bred with *Rhoa*^flox/flox^ mice to develop the chondrocyte-specific *Rhoa* conditional knockout mice (*Col2a1*^Cre−ERT^- *Rhoa*^flox/flox^, cKO), with littermate mice (*Col2a1*^Cre−ERT^, Cre) serving as controls (Fig. 3a and b). At 8 weeks of age, both cKO mice and Cre mice were was administered tamoxifen for continuously 5days, and subjected to prepare the OA model. In this model, Western blot and immunofluorescence staining revealed RhoA is hardly detected in the chondrocytes of the cKO group compared to the Cre group (Fig. 3c and d).

### 3.4 Chondrocytic *Rhoa* cKO alleviates the destruction of cartilage matrix in OA mice

To validate the surgical model and exclude genotype-specific baseline variations, sham-operated groups were established. Safranin O-fast green and Toluidine blue staining confirmed comparable cartilage morphology between sham-Cre and sham-cKO mice, while significant matrix degradation was observed in DMM groups relative to sham controls (Fig. S1a and b). These results verify successful OA induction and the specificity of the genetic manipulation, justifying focused comparison between DMM-Cre and DMM-cKO groups for assessing the therapeutic potential of RhoA deletion in OA progression.

**Fig. 4:**
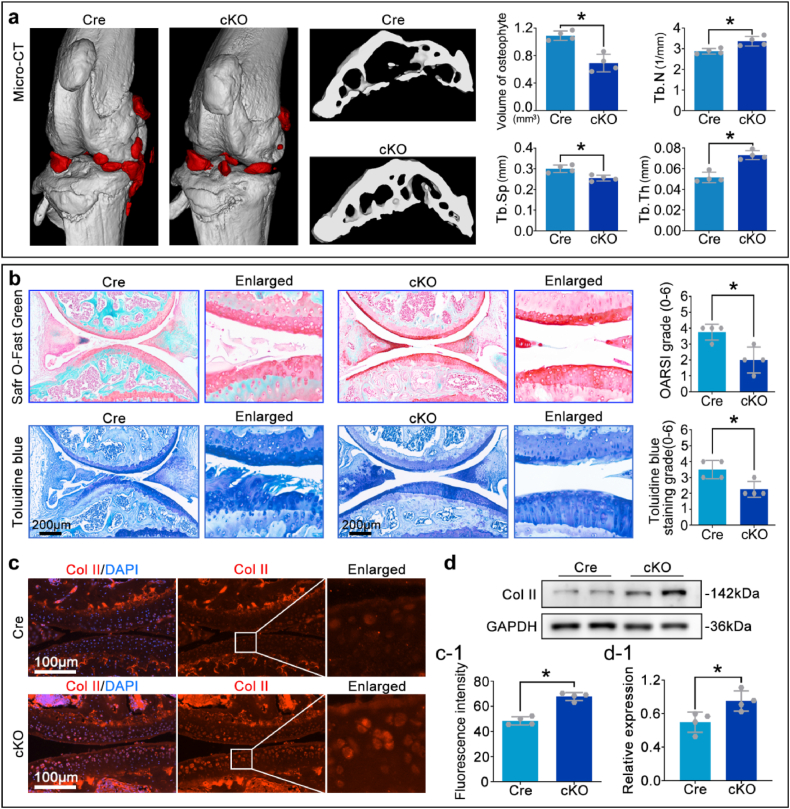
Loss of RhoA in chondrocytes alleviates cartilage matrix destruction in OA mice. **a.** Quantitative analysis of osteophyte volume and subchondral bone indicators (Tb.N, Tb.Th, Tb.Sp) through micro-CT three-dimensional reconstruction. **b.** Safranin O-fast green/Toluidine blue staining to assess cartilage matrix loss. **c, d.** Immunofluorescence staining and Western blot analysis of Collagen II fluorescence intensity and protein expression in cartilage tissue. ∗P < 0.05.

Eight weeks after OA modeling, micro-CT analysis revealed a significant reduction in osteophyte volume in the cKO group compared to the Cre group. In addition, the trabecular number (Tb.N) and trabecular thickness (Tb.Th) in the cKO group increased, and the trabecular separation (Tb.Sp) decreased (Fig. 4a). Subsequent paraffin section analysis, along with Safranin O-fast green and Toluidine blue staining, demonstrated a significantly higher cartilage matrix content in the cKO group compared to the Cre group (Fig. 4b). Immunofluorescence staining and Western blotting showed that the expression of Collagen II in cartilages layer was markedly increased in the cKO group (Fig. 4c and d).

### 3.5 Chondrocytic RhoA deficiency significantly reduced the proportion of fibrocartilage chondrocytes and inhibited the fibrotic lesions of cartilage tissue

**Fig. 5:**
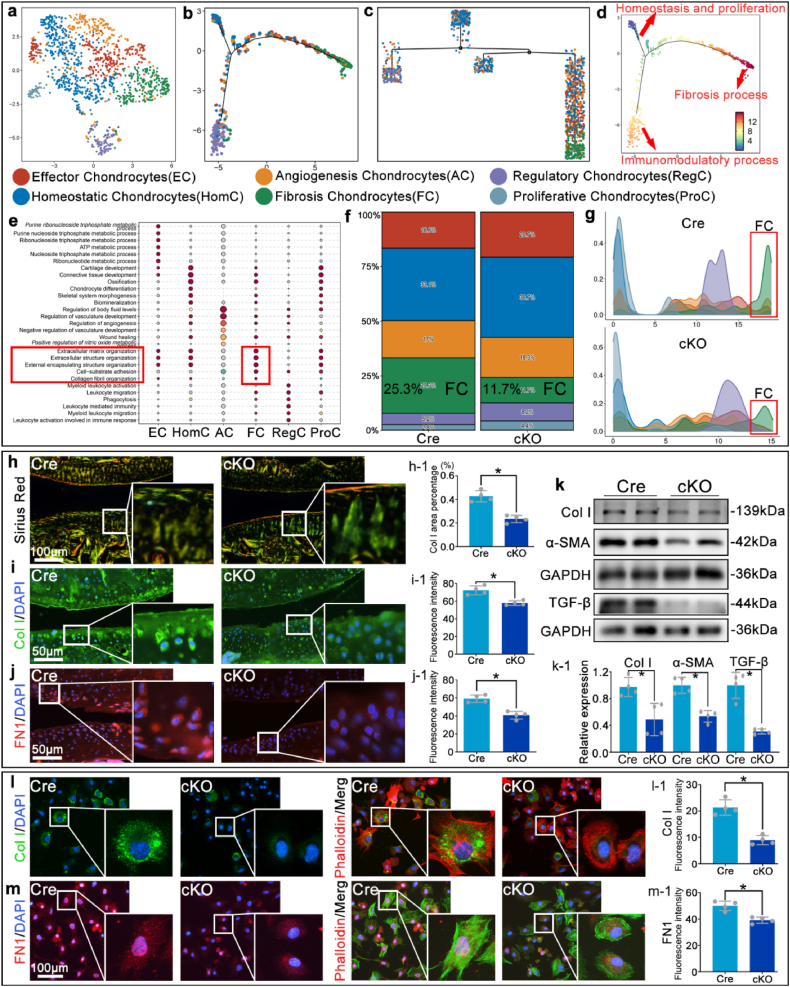
**RhoA deficiency in chondrocytes significantly inhibits cartilage fibrosis.** **a.** Single-cell sequencing of OA joint cartilage tissues from the Cre and cKO groups for subpopulation classification of chondrocytes. **b-c.** Perform time-series analysis using the Monocle2 algorithm. **d.** Use the CytoTRACE algorithm to analyze and display the degree of cell differentiation along each branch path of the proposed time-series. The trend from blue to red indicates that the degree of cell differentiation increases from low to high. **e.** GO functional analysis of each chondrocyte subpopulation to verify classification accuracy. **f.** Compare the proportions of each cartilage cell subpopulation in the Cre and cKO groups. **g.** The changes in cell density of each cartilage cell subpopulation in the branching path as depicted in Fig. 5b **h.** Sirius Red staining with polarized light microscopy (yellow/orange birefringence indicates type I collagen; green indicates type II collagen) showing reduced yellow/orange signal intensity in the cKO group compared to the Cre group. **i**, j**.** Immunofluorescence staining of Collagen I and Fibronectin 1 in cartilage tissue. k**.** Western blot detection of Collagen I, α-SMA, and TGF-β expression in cartilage tissue. **l, m.** Immunofluorescence analysis of primary chondrocytes isolated from Cre and cKO OA cartilage. **l.** Collagen I (green) and phalloidin-labeled F-actin (red). **m.** Fibronectin 1 (red) and phalloidin-labeled F-actin (green). ∗P < 0.05.

To investigate how RhoA regulates chondrocyte function and influences OA progression, joint cartilage tissues from OA mice in the Cre and cKO groups were extracted for single-cell sequencing. Chondrocytes were sequentially classified into 6 subgroups based on the expression profiles of specific marker genes and subsequent GO functional enrichment analysis (Fig. 5a–e, Fig. S2a–f). Temporal sequence analysis using Monocle2 algorithm revealed the distribution of different chondrocyte subtypes (Fig. 5b and c), and traced the progression of cells from the "steady state, proliferation" and "immune regulation process" to the "fibrotic process," suggesting that fibrosis may represent the terminal stage of chondrocyte fate (Fig. 5d). Bioinformatic fibrosis scoring, based on the aggregated expression of extracellular matrix organization genes and TGF-β family signaling, demonstrated markedly attenuated fibrotic signatures in the total chondrocyte population of the cKO group compared to Cre controls (Fig. S2g and h). GO functional enrichment analysis for each subpopulation confirmed the accuracy of the fibrocartilage chondrocytes classification (Fig. 5e). The analysis of cell subgroups revealed that the proportion of fibrocartilage chondrocytes in the cKO group was significantly decreased (Fig. 5f). The time-series analysis results showed that the number of chondrocytes transforming into fibrocartilage chondrocytes was reduced in the cKO group (Fig. 5b–g). Sirius Red staining revealed markedly reduced type I collagen fiber density and disorganized matrix architecture in the cKO group compared to the Cre group (Fig. 5h). Consistently, immunofluorescence staining and Western blot analysis revealed that the fluorescence intensity of fibrotic markers, including Collagen I and Fibronectin 1 [5], as well as the protein levels of Collagen I, α-SMA, and TGF-β, were significantly lower in the cartilage tissue of the cKO group compared to the Cre group (Fig. 5i–k). Primary chondrocytes isolated from Cre and cKO OA cartilage showed markedly reduced immunofluorescence intensity of both Collagen I and Fibronectin 1 in the cKO group. Phalloidin staining of F-actin further revealed a transition from the flattened, elongated cytoskeletal architecture in Cre cells to a contracted, rounded morphology in the cKO group (Fig. 5l and m).

### 3.6 RhoA regulates the expression of MMP2, thereby influencing the fibrosis of chondrocytes

**Fig. 6:**
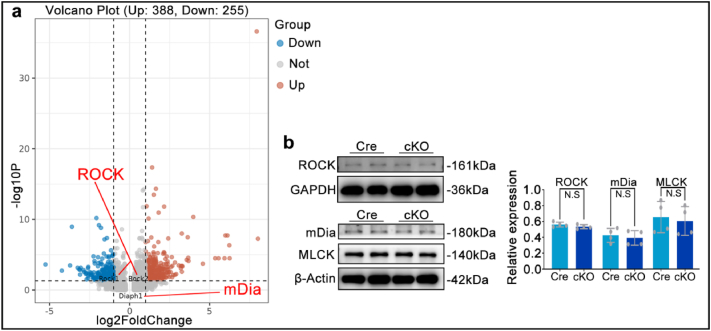
**a.** Single-cell sequencing analysis of ROCK and mDia expression levels in the fibrocartilage chondrocytes population from the Cre and cKO groups. **b.** Western blot detection of ROCK, mDia, and MLCK protein expression levels in cartilage tissue. N.S: no statistical difference.

To explore the regulatory mechanism of RhoA in chondrocyte fibrosis, the well-known classical downstream targets of RhoA were verified. Single-cell sequencing and Western blot analysis showed no significant changes in the expression of ROCK, MLCK, or mDia in the cKO group compared to the Cre group (Fig. 6a and b). These data indicates that condrocytic RhoA plays role in OA progression is independent on RhoA's conventional downstream pathways.

**Fig. 7:**
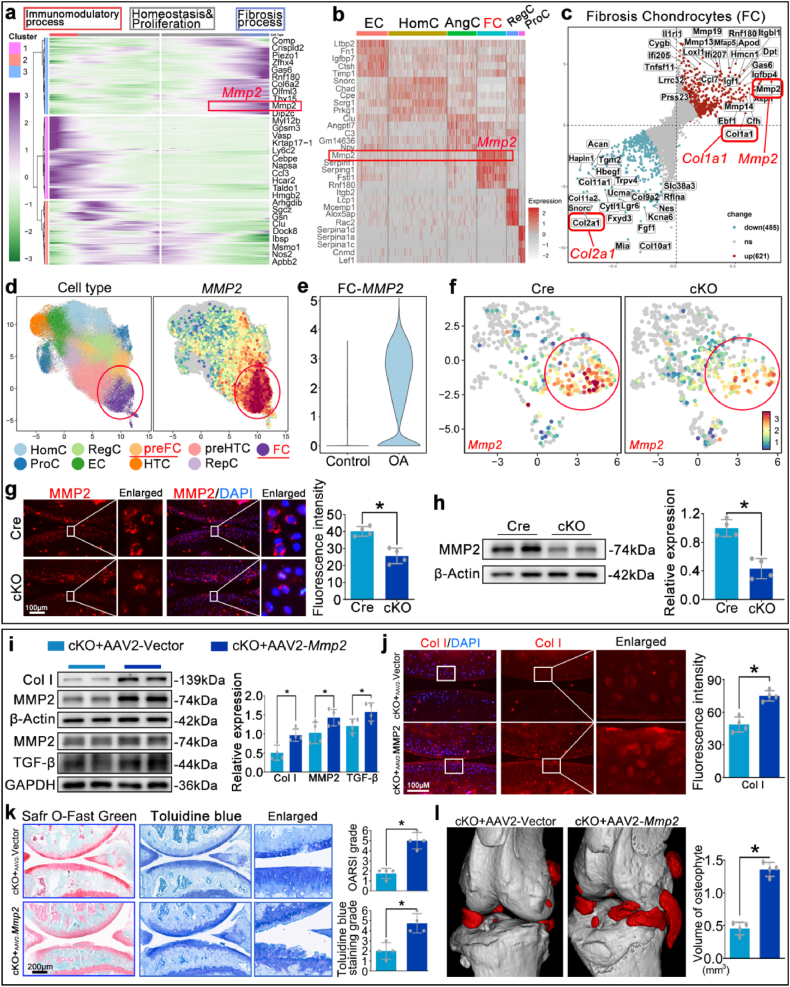
MMP2 mediates the role of chondrocytics RhoA in the cartilage fibrosis and OA progression. **a-b.** Temporal analysis of single-cell sequencing of Cre and cKO chondrocyte tissues, functionally enriching the gene trends regulating the transition from "immune regulation process" and "homeostasis, proliferation" to the "fibrosis process." **c.** Volcano plot analysis of single-cell sequencing data from Cre and cKO chondrocyte tissues, showing significant upregulation of *Mmp2* expression in the fibrocartilage chondrocytes subpopulation. **d, e.** Personalized analysis of the single-cell sequencing database from OA patient chondrocyte tissues, indicating that *Mmp2* is predominantly located in the OA fibrocartilage chondrocytes group (**d**), and is specifically expressed in OA fibrocartilage chondrocytes compared to the Control group (**e**). **f.** Differential expression volcano plot from single-cell sequencing showing *Mmp2* expression status. **g, h.** Immunofluorescence staining and Western blot detection of MMP2 expression in chondrocyte tissues. **i, j.** Western blot and Immunofluorescence staining detection of Collagen I expression in the cKO + AAV2-Vector group and cKO + AAV2-*Mmp2* group. **k.** Safranin O-fast green/Toluidine blue staining to detect cartilage matrix loss. **l.** Micro-CT quantitative analysis of osteophyte volume. ∗P < 0.05.

Therefore, the Monocle2 algorithm assay was conducted to analyze the differential expression genes (DEGs) on the mice single-cell sequencing data. By combining the FindAllMarkers algorithm for differential analysis, it was discovered that during the process of normal chondrocytes transforming into fibrocartilage chondrocytes, the *Mmp2* gene was significantly upregulated (Fig. 7a–c). Notably, analysis on the single-cell sequencing database of OA patients also confirmed that *Mmp2* was highly expressed in the fibrocartilage chondrocytes population (Fig. 7d and e). Further bioinformatic analysis revealed that *Mmp2* was predominantly enriched in the fibrocartilage chondrocytes sub-population, with a significant reduction in the number of *Mmp2*-positive cells in the cKO group compared to the Cre group (Fig. 7f).

To further validate the targeting effect of MMP2, the OA cartilages from the Cre and cKO groups were extracted for whole-tissue transcriptome sequencing analysis (Fig. S4a–e). We then conducted an association analysis between the significantly DEGs sets in these tissues and the gene sets highly expressed during the fibrosis process enriched by single-cell sequencing (Cluster3). This resulted in 56 intersecting genes (Fig. S4f). Using the STRING database, protein-protein interaction analysis suggested potential associations among RhoA, MMP2, and the key fibrosis factor TGF-β (Fig. S4g). Subsequent immunofluorescence staining and Western blot confirmed that MMP2 expression in OA cartilage tissue and cells from the cKO group was significantly lower than in the Cre group (Fig. 7g and h), consistent with its predicted role as a downstream node of the RhoA axis.

To determine whether RhoA affects cartilage fibrosis and influences OA progression by targeting MMP2, an *Mmp2* overexpression virus (AAV2-*Mmp2*) was constructed. Western blot and Immunofluorescence staining further demonstrated that, compared to the cKO group, the cartilage fibrosis index, as indicated by Collagen I, was significantly increased in the cKO + AAV2-*Mmp2* group (Fig. 7i and j). Safranin O-fast green/Toluidine blue staining and Micro-CT revealed that the AAV2-*Mmp2* treatment significantly reversed the inhibitory effect of *Rhoa*-cKO on osteophyte formation and its protective effect on the cartilage matrix (Fig. 7k and l). To further validate this signaling pathway in a loss-of-function context as suggested, we established an in vitro fibrosis model using wild-type primary chondrocytes stimulated with IL-1β [5]. siRNA-mediated knockdown of *Mmp2* significantly attenuated IL-1β-induced Collagen-I upregulation at the protein level. Consistently, immunofluorescence staining further confirmed reduced fluorescence intensity of both Collagen I and Fibronectin 1 in knockdown cells compared to IL-1β-treated controls (Fig. S3a–c).

### 3.7 RhoA regulates the expression of MMP2 through the β-catenin/SOX4 axis

**Fig. 8:**
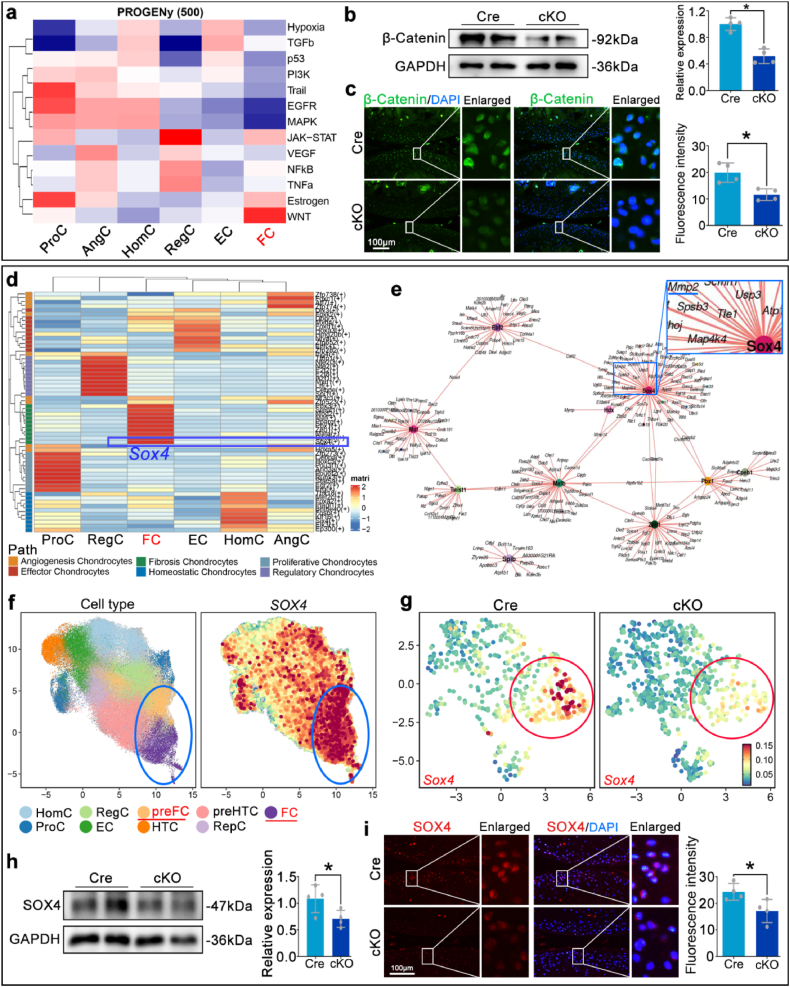
The β-Catenin/SOX4 axis modulates the regulating of MMP2 expression by RhoA. **a.** Sequencing results of cartilage single cells from Cre and cKO mice, showing significant WNT signaling pathway activity in the fibrocartilage chondrocytes population. **b, c.** Immunofluorescence staining and Western blot analysis to detect β-Catenin expression in the cartilage tissues of Cre and cKO mice. **d.** Analysis of transcription factors in each cell subgroup of Cre and cKO mouse cartilage sequencing. **e.** TF-Gene regulatory network analysis of transcription factors and gene regulatory networks in the top 10 subpopulations within the fibrocartilage chondrocytes group. **f.** Personalized analysis of *SOX4* expression in each cartilage cell subgroup using the single-cell sequencing database of OA patient cartilage tissues. **g.** Analysis of the cellular localization and expression levels of *Sox4* in Cre and cKO mouse cartilage single cells. **h, i.** Immunofluorescence staining and Western blot detection of SOX4 expression in the cartilage tissues of Cre and cKO mice. ∗P < 0.05.

To explore the molecular mechanism by which RhoA regulates MMP2, single-cell sequencing data from OA mice were analyzed using the PROGENy algorithm. The results revealed that the WNT signaling pathway was the most active in the fibrocartilage chondrocytes population (Fig. 8a). Western blotting and immunofluorescence staining results showed significantly lower protein levels and fluorescence intensities of β-Catenin, the key effector molecule of the WNT pathway, in the cKO group compared to the Cre group (Fig. 8b and c). Further using the SCENIC algorithm to analyze the transcription factors of each chondrocyte subpopulation in Cre and cKO groups, we identified the top 10 transcription factors highly expressed in the fibrocartilage chondrocytes (Fig. 8d). TF-Gene regulatory network analysis indicated that *Sox4*, ranked first, directly regulates *Mmp2* (Fig. 8e). Based on previous literature [30], SOX4 might be a key factor connecting β-Catenin and MMP2. Personalzsed analysis on the single-cell sequencing database of OA patient confirmed the significant expression of *SOX4* in the fibrocartilage chondrocytes population, suggesting that *SOX4* has potential as a therapeutic target for this subgroup (Fig. 8f, Fig. S5a–d). Further examination of single-cell sequencing data from OA mouse cartilage tissue revealed that *Sox4*-positive cells were primarily localized to the fibrocartilage chondrocytes population in Cre mice, with a notable reduction in *Sox4* expression in the cKO group (Fig. 8g). Immunofluorescence staining and Western blot analysis confirmed that the protein levels of SOX4 in the cKO group were significantly lower than in the Cre group (Fig. 8h and i). These results suggest that β-Catenin/SOX4 axis modulates the regulating MMP2 expression by RhoA.

**Fig. 9:**
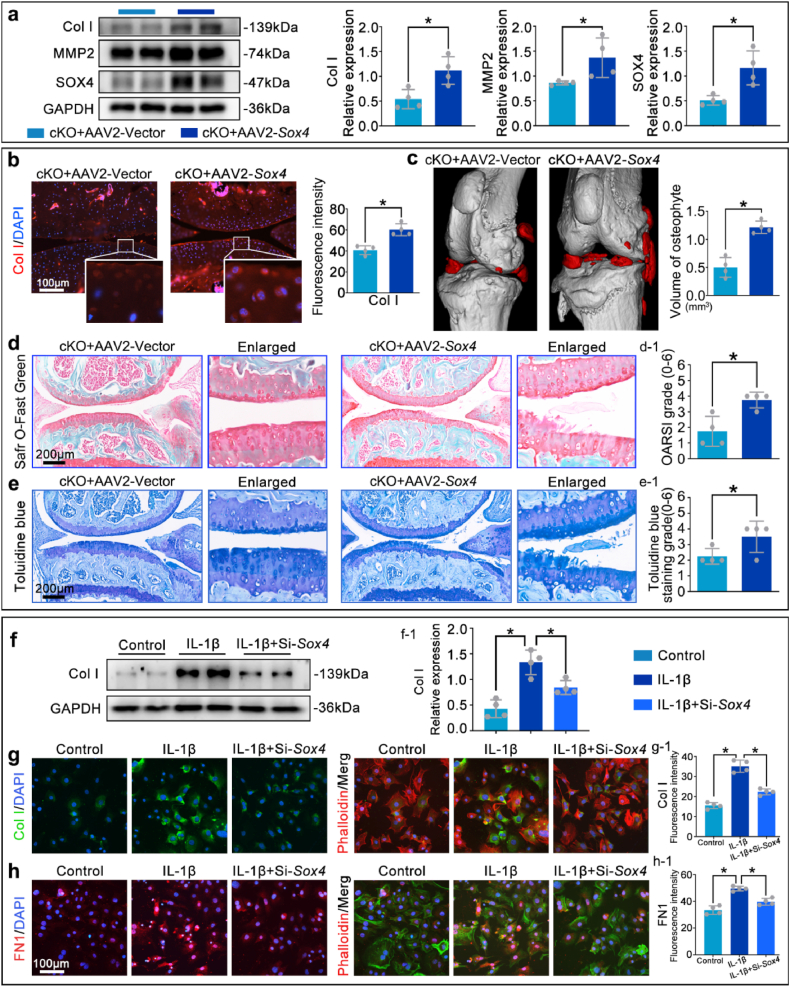
Overexpression of *Sox4* significantly reversed the inhibitory effect of *Rhoa*-cKO on fibrosis and increased cartilage matrix degradation. **a.**Western blot detection of MMP2, SOX4, and Collagen I protein expression levels in cartilage tissue. **b.** Immunofluorescence staining to detect changes in Collagen I fluorescence intensity in cartilage tissue. **c.** Quantitative analysis of osteophyte volume changes using Micro-CT. **d, e.** Detection of cartilage matrix loss using Safranin O-fast green and Toluidine blue staining. **f.** Western blot analysis of Collagen I expression in primary chondrocytes treated with control medium, IL-1β, or IL-1β+Si-*Sox4*. **g, h.** Immunofluorescence analysis of primary chondrocytes under indicated treatments: **g.** Collagen I (green) and phalloidin-labeled F-actin (red). **h.** Fibronectin 1 (red) and phalloidin-labeled F-actin (green). ∗P < 0.05.

To further validate above mechanism, an intra-articular injection of *Sox4*-overexpressing virus (AAV2-*Sox4*) was executed. Western blots and immunofluorescence staining demonstrated that, compared to the cKO group, the expression levels of SOX4, MMP2, and Collagen I were significantly increased in the cKO + AAV2-*Sox4* group (Fig. 9a and b). Micro-CT and Safranin O-fast green/Toluidine blue staining showed that the AAV2-*Sox4* treatment significantly reversed the inhibitory effect of *Rhoa*-cKO on osteophyte formation and the protective effect on cartilage matrix (Fig. 9c–e). Conversely, knockdown of *Sox4* by siRNA in IL-1β-stimulated primary chondrocytes markedly reduced Collagen I and Fibronectin 1 expression, mirroring the opposite effects observed in the AAV2-*Sox4* overexpression experiments (Fig. 9f–h).

## 4 Discussion

RhoA has been implicated in tissue repair across various systems, including the skin, myocardium, and nervous tissue [[31], [32], [33], [34]]. However, its role and mechanisms in OA, particularly in chondrocytes of OA, remain poorly understood. To date, no study has investigated its specific role in OA by modulating RhoA expression or activation in chondrocytes.

Accumulating evidence indicates that RhoA signaling contributes to fibrogenesis across multiple organs, including the heart, lung, liver, kidney, and skin. Mechanistically, these studies reveal diverse downstream pathways: RhoA/ROCK-mediated cytoskeletal remodeling and cell contraction in cardiac and pulmonary fibroblasts, metabolic reprogramming via glycolysis or fatty acid oxidation inhibition in dermal and cardiac tissues, post-translational regulation of protein stability in renal fibrosis, and mitochondrial dynamics control through YAP signaling [[35], [36], [37], [38]]. Given the chondrocyte fibrosis plays a crucial role in OA progression, making sure the specific role and mechanism of RhoA in chondrocytes, which are still unknown, might help us to understand the pathology of OA and to explore novel therapeutic strategy for clinical treatment. Thus, this study integrated the bio-informatic analysis on human and mice OA single-cell sequencing database, as well as the chondrocytic *Rhoa* cKO mice, and then identified the significant upregulation of RhoA in OA chondrocytes, and RhoA deficiency in chondrocytes was shown to delay cartilage fibrosis and the degradation of the cartilage matrix.

Cartilage fibrosis is widely recognized as the central process in the irreversible degradation of the cartilage matrix during OA [39]. However, the pathological mechanisms underlying cartilage fibrosis differ from general tissue fibrosis, and the initiation of this process remains a subject of debate in the scientific community. Multiple pathways, including TGF-β/Smad and Wnt/β-catenin, have been implicated in fibrosis, but their interrelationships and the upstream initiating factors in OA cartilage fibrosis remain poorly understood. Through single-cell sequencing analysis in OA patients cartilage, the "hsa04810: Regulation of actin cytoskeleton" was enriched in the fibrocartilage chondrocytes subpopulation. And RhoA, a well known key molecule in regulating the dynamics of actin cytoskeleton, emerges as the most highly up-regulated key regulatory factors in the actin cytoskeleton pathway. These findings indicate RhoA mediated actin cytoskeletal rearrangement plays important role in the OA cartilage fibrosis. And this view was verified by a series experimental data with chondrocyte specific RhoA cKO.

It is particularly worth noting that, this study demonstrated that the chondrocytic RhoA regulating cartilage fibrosis and OA progression is independent on RhoA's conventional downstream pathway. The traditional view generally posits that RhoA regulates various processes, such as cytoskeletal rearrangement, cell migration, and inflammatory responses, primarily through its classical downstream target, ROCK or mDia [40,41]. However, in this study, the expression of ROCK and mDia, as well as their downstream molecule MLCK did not show significant differences between the *Rhoa* cKO group and Cre group. This suggests any unknown pathway is involved in RhoA's role in chondrocyte. Recently, there are several studies demonstrated that RhoA plays role in some cells through different unconventional pathways. For instance, in breast cancer, RhoA phosphorylates the Y731 site of VE-cadherin, disrupting its adhesive function and promoting invasion and metastasis [42]. RhoA has also been shown to regulate Schwann cell proliferation and differentiation through AKT or JNK pathways rather than the ROCK pathway [23,43]. These findings can help us to understand why the clinic trials of ROCK inhibitors (e.g., Y27632 or Fasudil) may not accurately simulate or block RhoA's role in specific pathological contexts. Similarly, this study highlights that targeting the classical downstream molecules of RhoA is not an effective strategy for alleviating chondrocyte fibrosis in OA tissues. To systematically elucidate RhoA's specific mechanism, single-cell sequencing, tissue transcriptome sequencing, and functional rescue experiments were integrated, ultimately identifying MMP2 as the core downstream effector molecule driving RhoA-induced fibrosis. In the context of OA pathology, MMP2 is not only a key matrix-degrading enzyme, it also mediates cartilage matrix degradation, amplifies the inflammatory response, and regulates chondrocyte proliferation and apoptosis [44,45]. Additionally, MMP2 emerges as a specific biomarker for the fibrocartilage chondrocyte subpopulation, highlighting its distinct association with the fibrotic subset in OA cartilage [12]. Notably, previous studies have established MMP2 as a critical pathogenic mediator and therapeutic target across multiple fibrotic diseases, including pressure overload-induced cardiac fibrosis, renal fibrosis, and idiopathic pulmonary fibrosis, further supporting its position as a pivotal node in fibrotic disease [[46], [47], [48]]. Moreover, although MMP2 overexpression correlated with elevated TGF-β levels and potential reciprocal regulation has been documented, given the pleiotropic, concentration-dependent roles of TGF-β in both cartilage homeostasis and fibrosis, we interpret these changes as accompanying phenotypic alterations, positioning TGF-β as a reference index rather than a confirmed downstream target [49]. Building on previous findings—such as Xia et al.’s confirmation that extracellular matrix mechanical signals are regulated by the RhoA-β-Catenin axis in inner ear progenitor cell expansion and Fu et al.’s discovery of the WNT/β-Catenin-SOX4 positive feedback loop driving gastric cancer drug resistance and progression [30,50]—this study conducted experiments focused on the upstream and downstream molecular mechanisms of MMP2. These experiments ultimately revealed that RhoA drives chondrocyte fibrosis through a previously unreported non-classical β-Catenin/SOX4/MMP2 signaling axis.

From a translational perspective, RhoA pathway inhibitors (such as AH001, JTE-013, and ATL-III) have shown significant efficacy in heart failure, pulmonary fibrosis, and myocardial fibrosis [26,36,51], suggesting broad clinical translational potential. However, recent studies indicate RhoA's functions and mechanisms vary significantly across different tissues and cell types. For example, a study by Stern et al. demonstrated that conditional *Rhoa* knockout in neurons and astrocytes promotes and inhibits spinal cord injury repair, respectively [52]. Within the joint organ, studies have shown that RhoA activation in subchondral bone endothelial cells induces ferroptosis and disrupts vascular endothelial cadherin distribution. In contrast, recent investigations have revealed that RhoA mediates protective functions in synovial macrophages through the Hippo/YAP/CCN2 axis [17,21]. Extending these findings, the present study identifies that RhoA conversely drives fibrogenesis in chondrocytes via the SOX4/MMP2 cascade. Therefore, our findings and literature analysis indicate that, broad application of RhoA inhibitors in OA is therapeutically undesirable, as it would compromise protective macrophage functions while aiming to suppress chondrocyte fibrosis. Instead, chondrocyte-specific targeting circumvents the cell-type-specific heterogeneity of RhoA across joint tissues, thereby maximizing therapeutic efficacy to better delay OA progression.

## 5 Conclusions

This study is the first to demonstrate that RhoA expression is significantly upregulated in chondrocytes during the development and progression of both human and mouse OA. Conditional gene knockout mice revealed that *Rhoa* deficiency specifically inhibits chondrocyte fibrosis and slows OA progression, independent of the classical ROCK or mDia pathway. Instead, this effect is mediated by the non-classical β-Catenin/SOX4/MMP2/TGF-β axis. These findings not only clarify the cell type-specific role of RhoA in OA pathogenesis but also provide experimental support for targeting RhoA or its downstream molecules as a therapeutic strategy for OA. Further research is necessary to examine the dynamic regulation of this signaling axis in chondrocytes at different stages and to assess its potential for clinical intervention.

## Author contributions

Yizhou Xu: Conceptualization, Formal analysis, Funding acquisition, Investigation, Methodology, Writing–original draft, Writing–review & editing. Shuyi Xu: Conceptualization, Formal analysis, Investigation, Methodology, Writing – original draft, Writing–review & editing. Jiayi Li: Writing–review & editing, Methodology, Validation. Shicheng Wang: Conceptualization, Methodology, Writing–review & editing, Validation. Jie Liang: Investigation, Validation. Jiaqi Wang: Investigation, Validation. Xianghai Wang: Investigation, Validation. Gang Deng: Resources, Supervision, Funding acquisition. Lixin Zhu: Writing – review & editing, Project administration, Supervision. Jiasong Guo: Conceptualization, Writing – review & editing, Project administration, Supervision.

## Declarations ethics approval and consent to participate

The observational study involving clinical samples in this study was conducted in accordance with the ethical principles outlined in the Declaration of Helsinki and was approved by the Ethics Committee of Ganzhou Hospital (Approval number: TY-ZKY2024-141-01, date: 10/20/2024). All procedures involving human samples were designed to respect the rights, dignity, and privacy of participants. Informed consent was obtained from all participants, or their legally authorized representatives. The specimens collected in this study were from tissues that had to be removed during surgery for therapeutic needs, so that participants did not have potential risks and burdens. All animal use in this study was approved by the Ethics Committee of Southern Medical University. All of the experiments were performed in accordance with the Basel Declaration.

## Consent for publication

All authors have thoroughly reviewed this manuscript and kindly request its exclusive consideration for publication.

## Declaration of generative AI in scientific writing

No generative artificial intelligence (AI) or AI-assisted technologies were used in the preparation of this manuscript.

## Funding

The author(s) disclose receipt of the following financial or material support for the research, authorship, and/or publication of this article: this work was supported by the 10.13039/501100002858China Postdoctoral Science Foundation (2024M761333); Key Research and Development Project of Ganzhou, Jiangxi Province (2023LNS17445).

## Declaration of competing interest

The authors declare that they have no known competing financial interests or personal relationships that could have appeared to influence the work reported in this paper.

## Data Availability

The data used to support the findings of this study are available from the corresponding author upon request.
