# Supplementary material for: RhoA deficiency in chondrocyte inhibits cartilage fibrosis and ameliorates osteoarthritis progression via SOX4/MMP2 axis
Source: J Orthop Translat. 2026 May 14;58:101127. doi: 10.1016/j.jot.2026.101127 (PMC13206727; doi:10.1016/j.jot.2026.101127)

**Figure-2c**


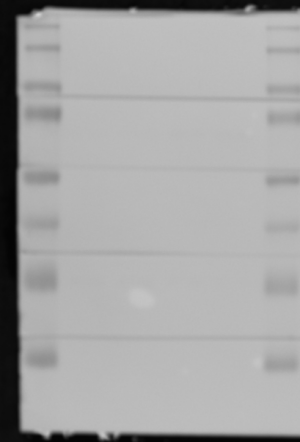

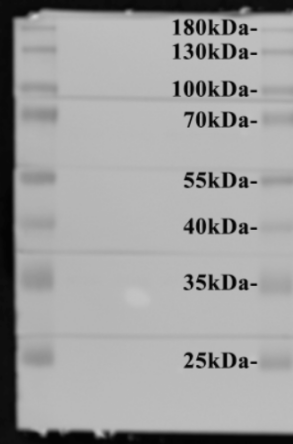

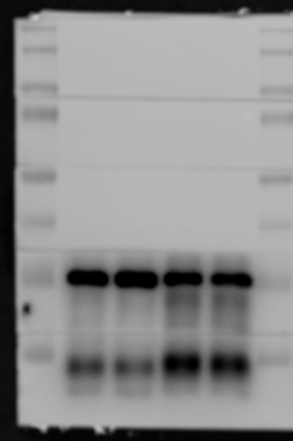

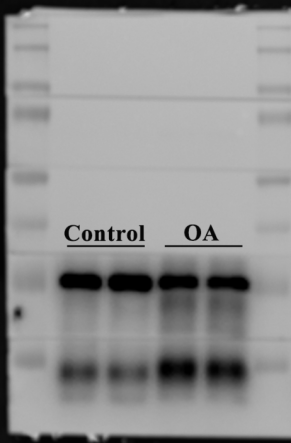


**Figure-2e**


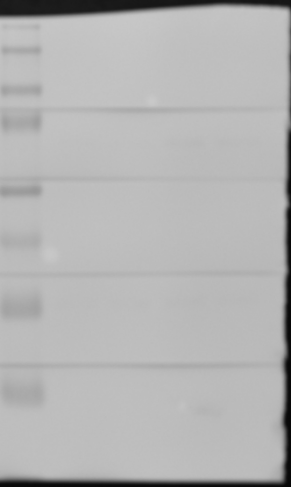

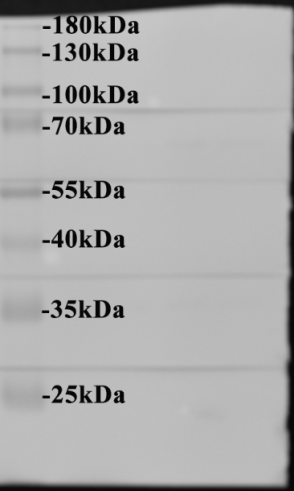

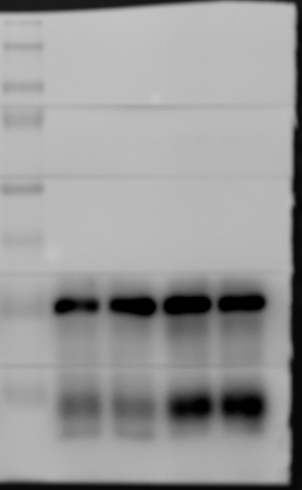

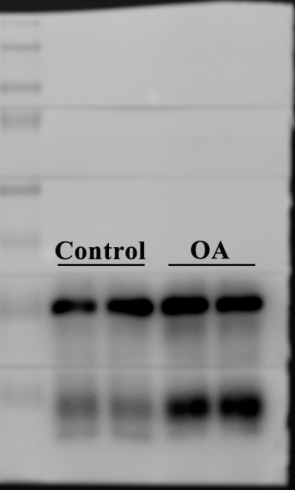


**Figure-3c**


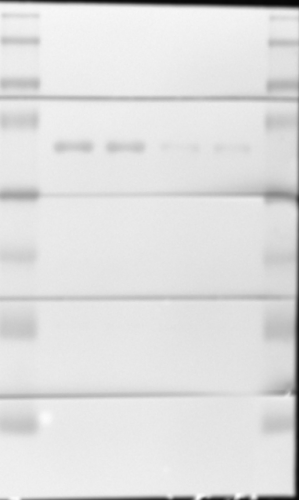

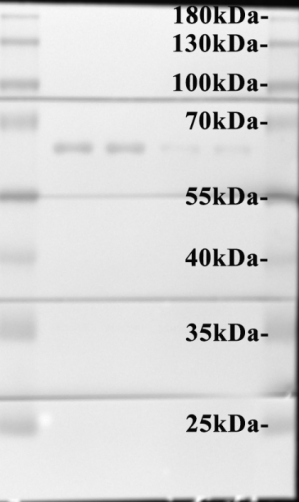

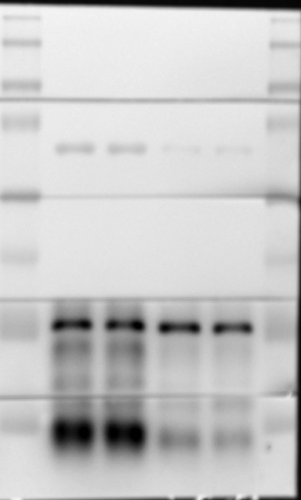

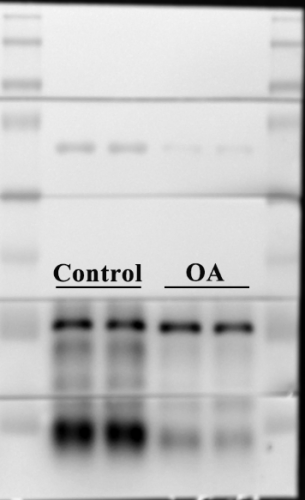


**Figure-4c**


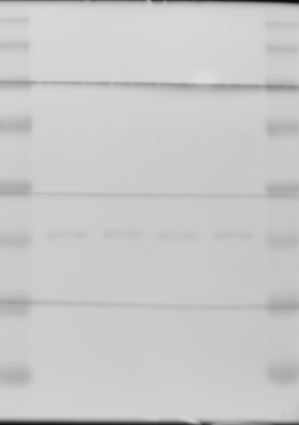

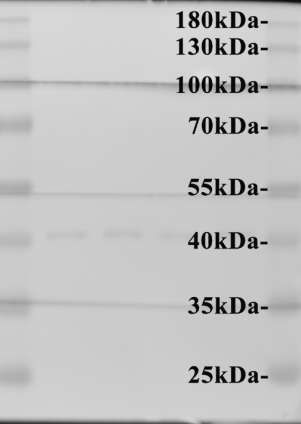

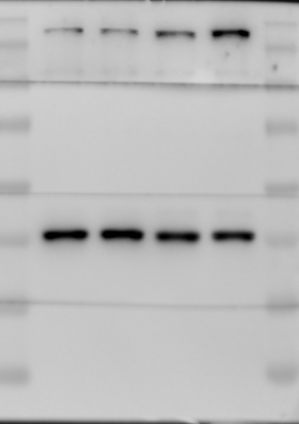

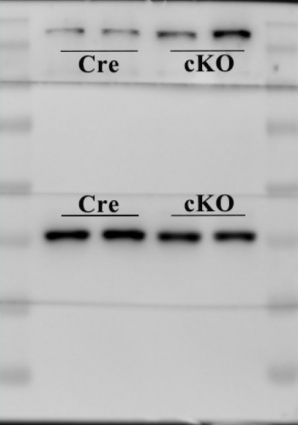


**Figure-5k**


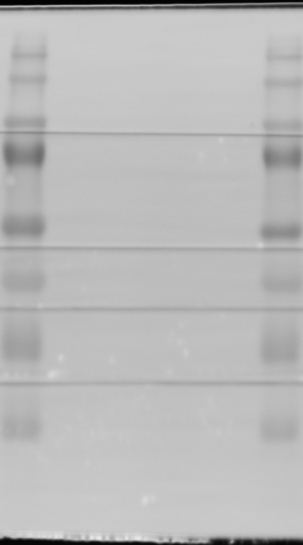

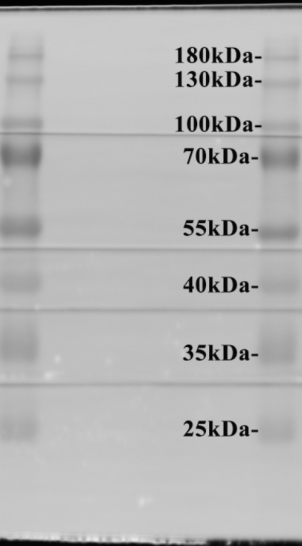

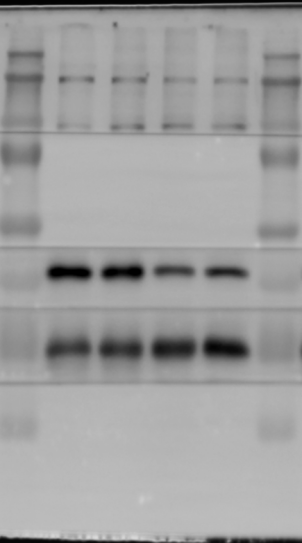

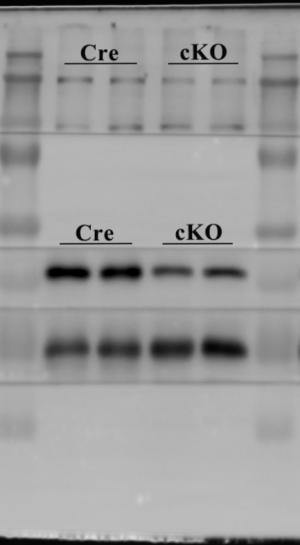


**Figure-5k**


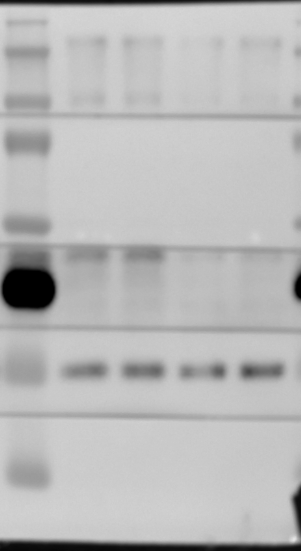

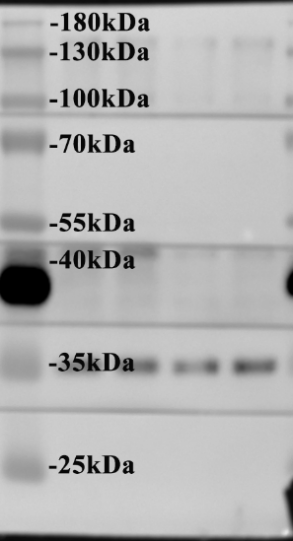

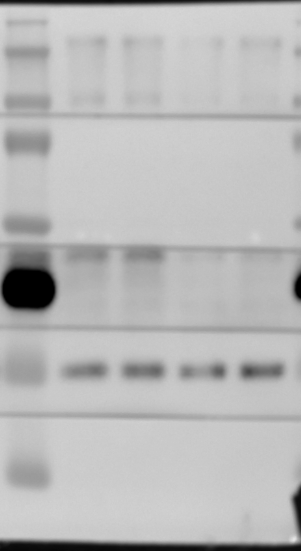

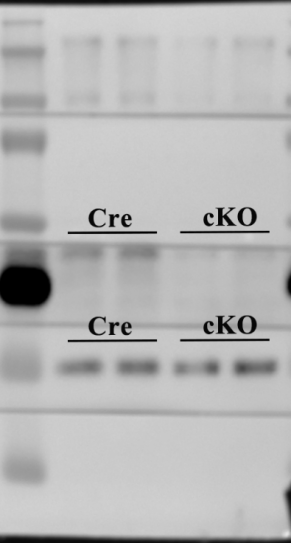


**Figure-6b**


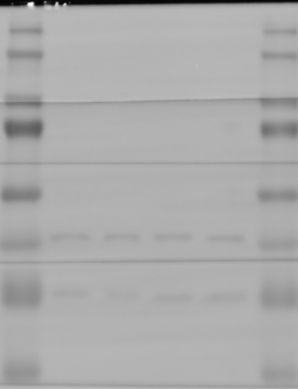

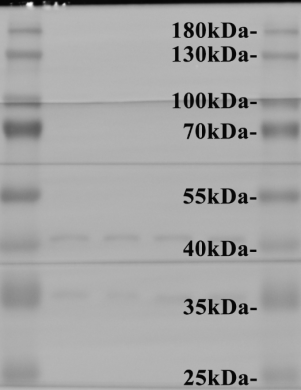

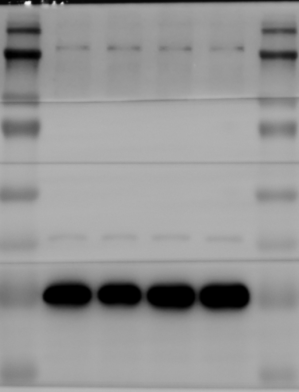

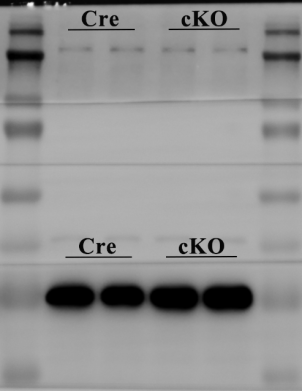


**Figure-6b**


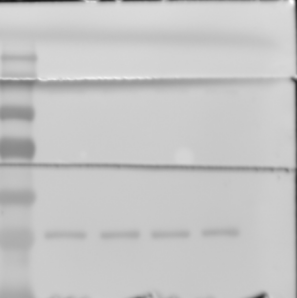

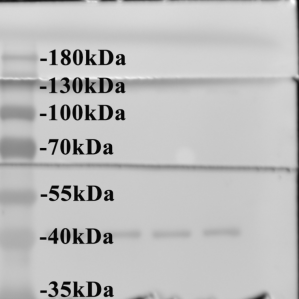

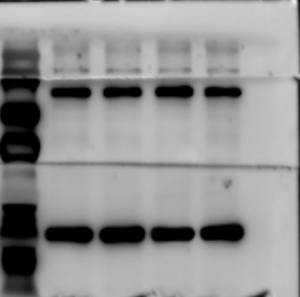

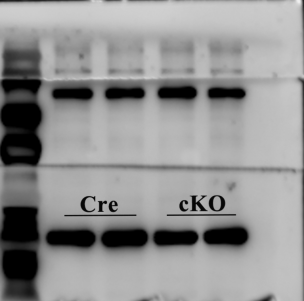


**Figure-7h**


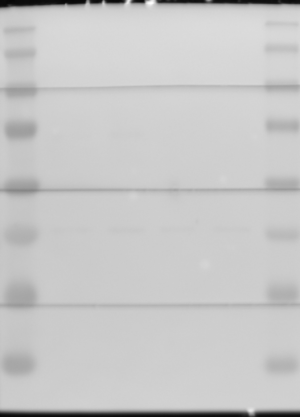

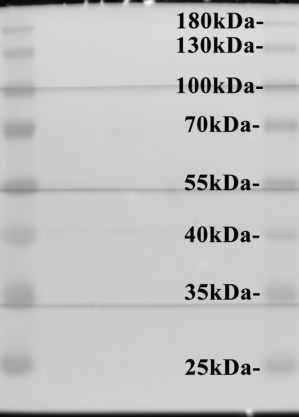

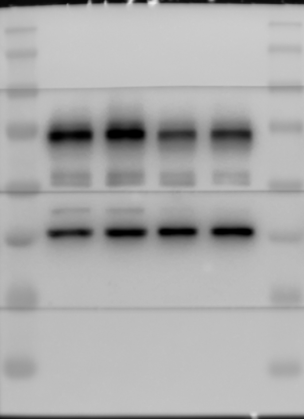

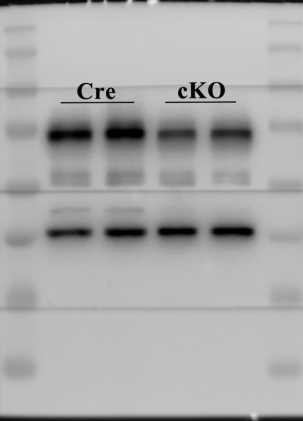


**Figure-7i**


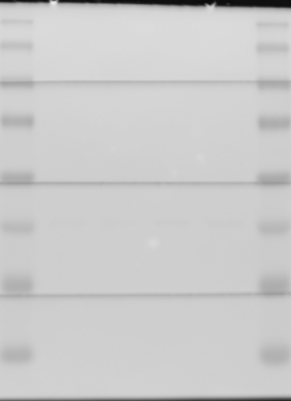

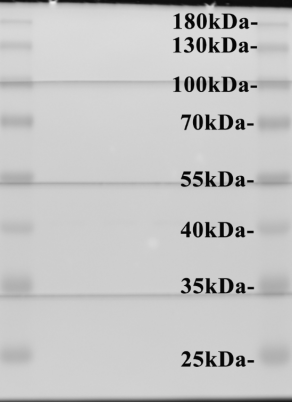

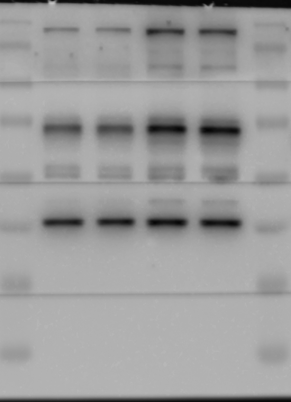

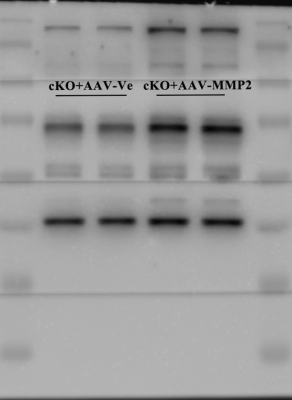


**Figure-8b**


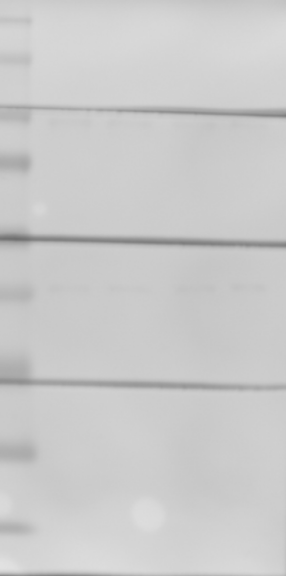

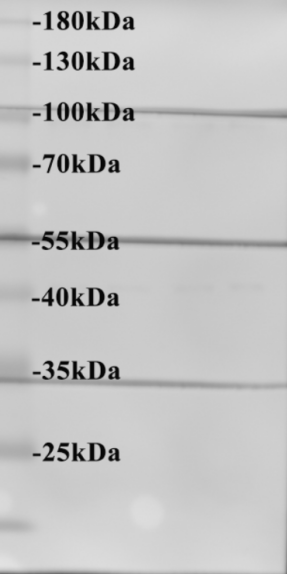

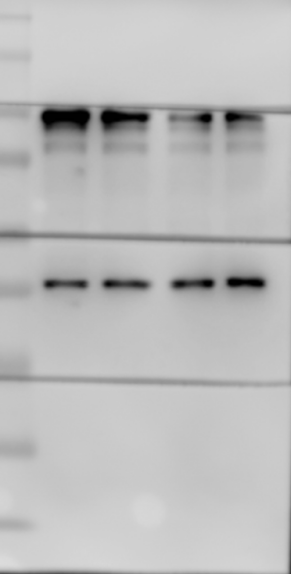

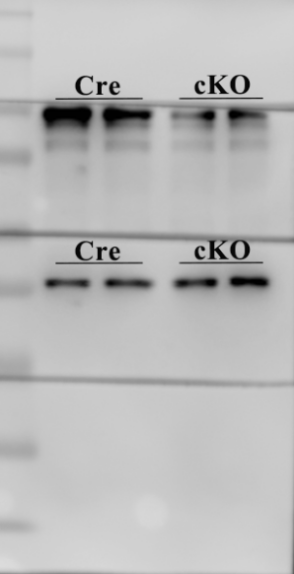


**Figure-8h**


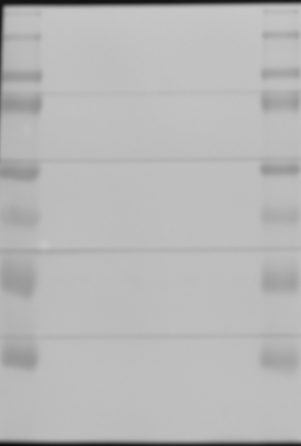

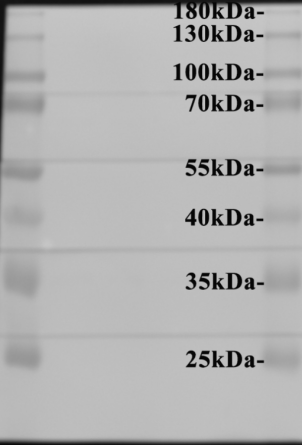

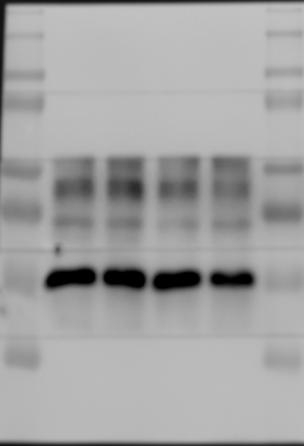

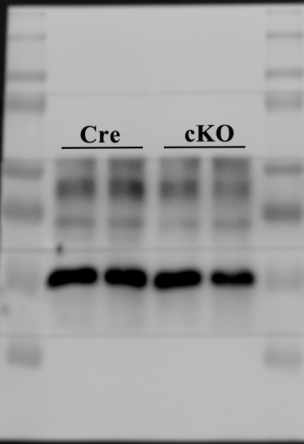


**Figure-9a**


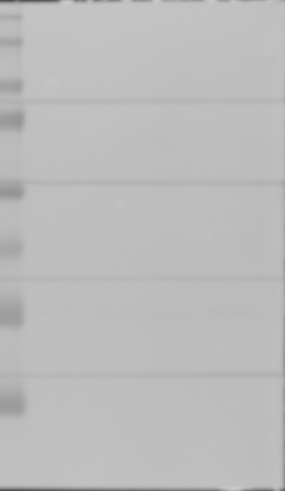

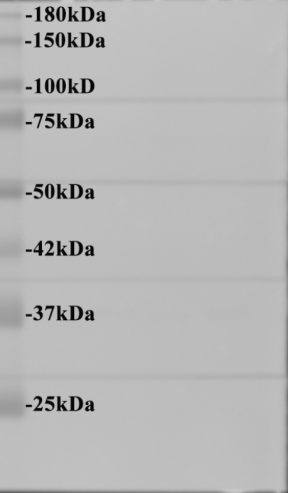

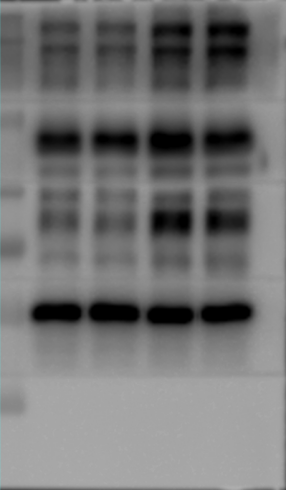

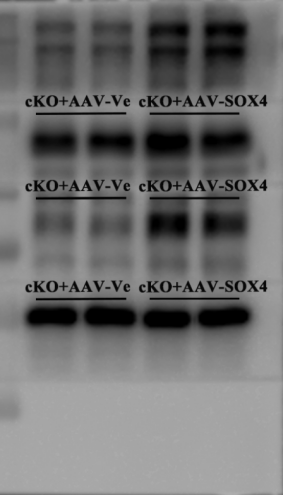


**Figure-9f**


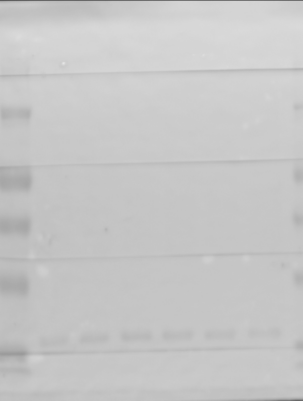

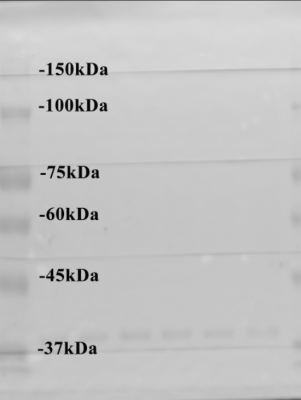

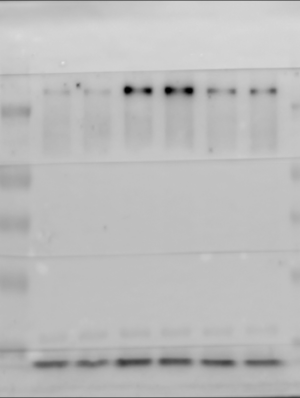

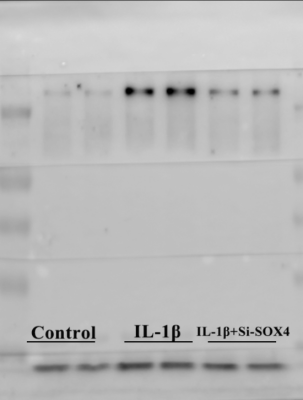


**Figure-S3a**


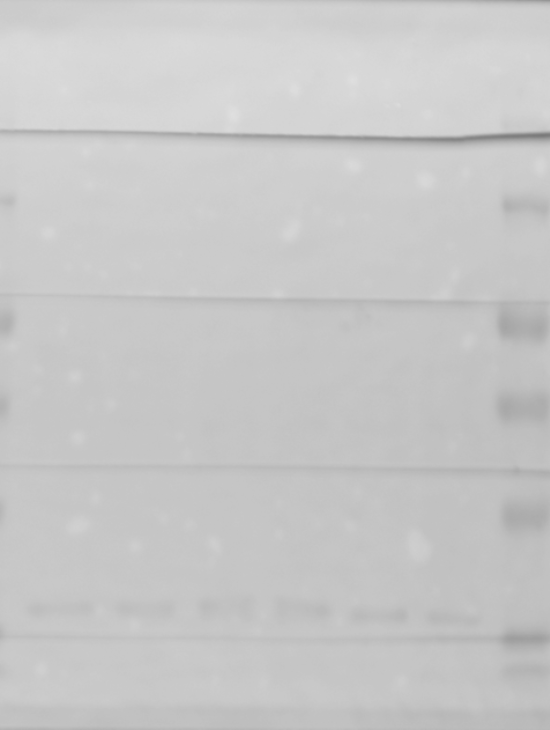

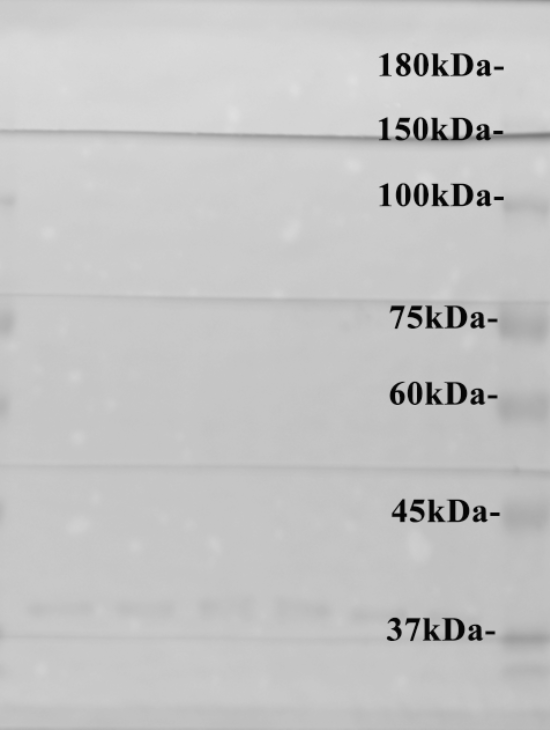

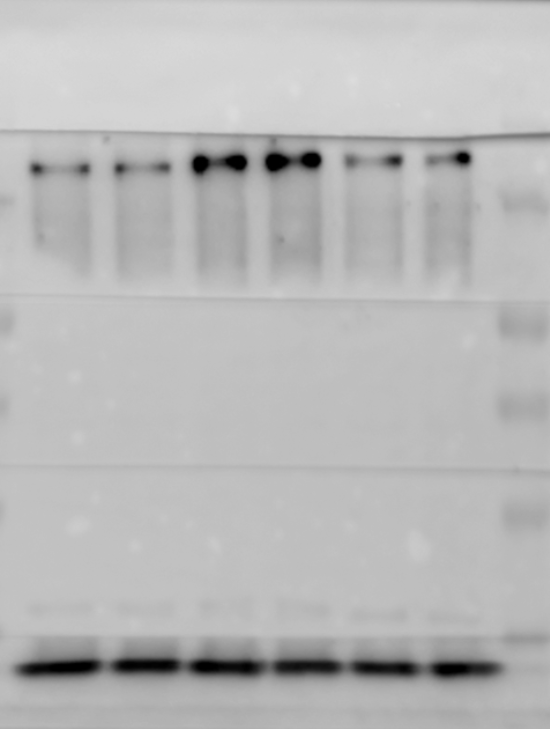

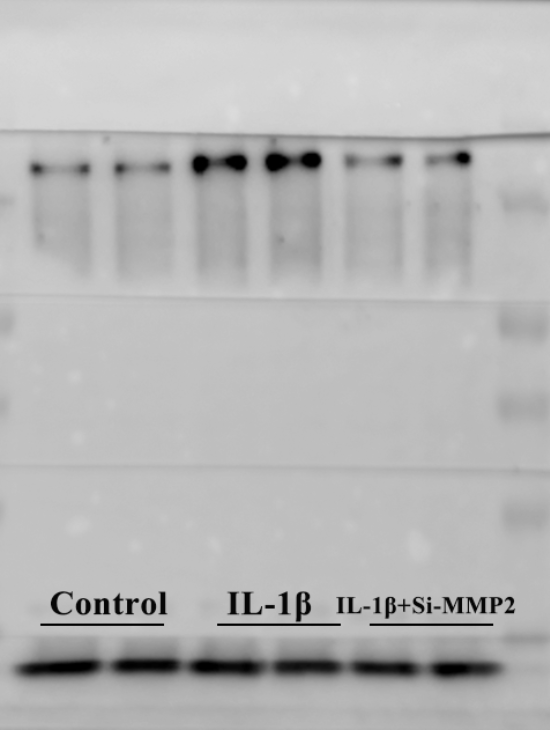

Supplement: Multimedia component 1 [file mmc1.docx]
