# Supplementary material for: RhoA deficiency in chondrocyte inhibits cartilage fibrosis and ameliorates osteoarthritis progression via SOX4/MMP2 axis
Source: J Orthop Translat. 2026 May 14;58:101127. doi: 10.1016/j.jot.2026.101127 (PMC13206727; doi:10.1016/j.jot.2026.101127)
Supplement: Multimedia component 2 [file mmc2.docx]

**Supplement-Figure-1**


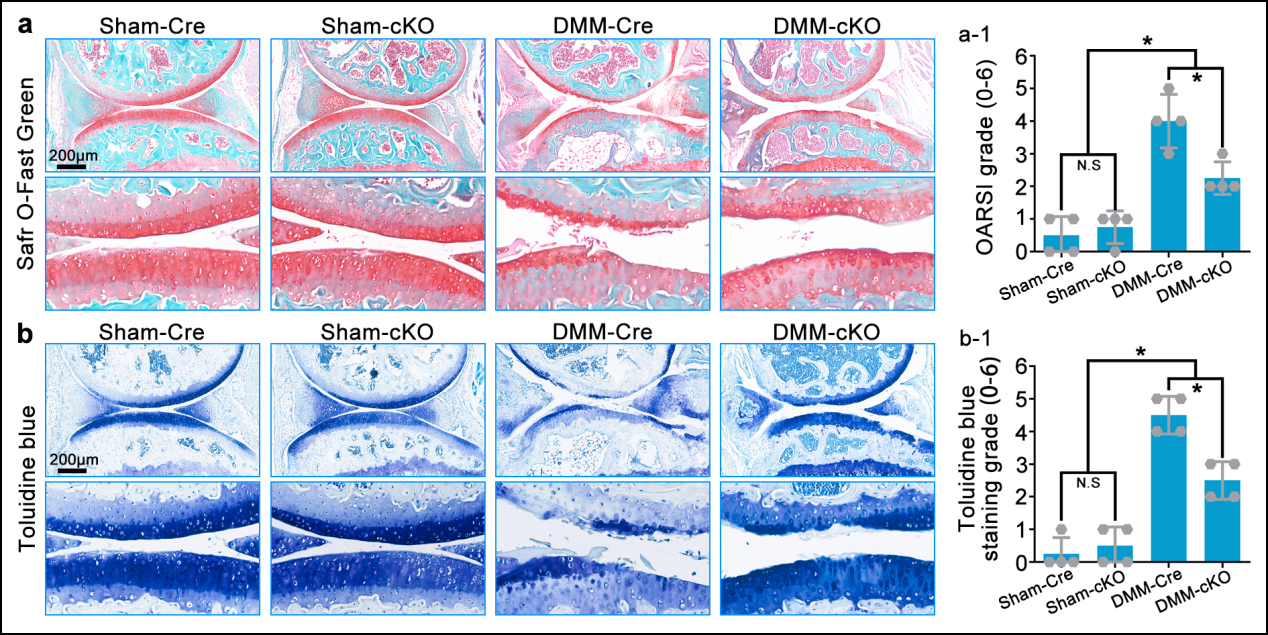


**Supplement-Figure-1: a**, **b.** Safranin O-fast green/Toluidine blue staining to assess cartilage matrix loss. *P < 0.05.

**Supplement-Figure-2
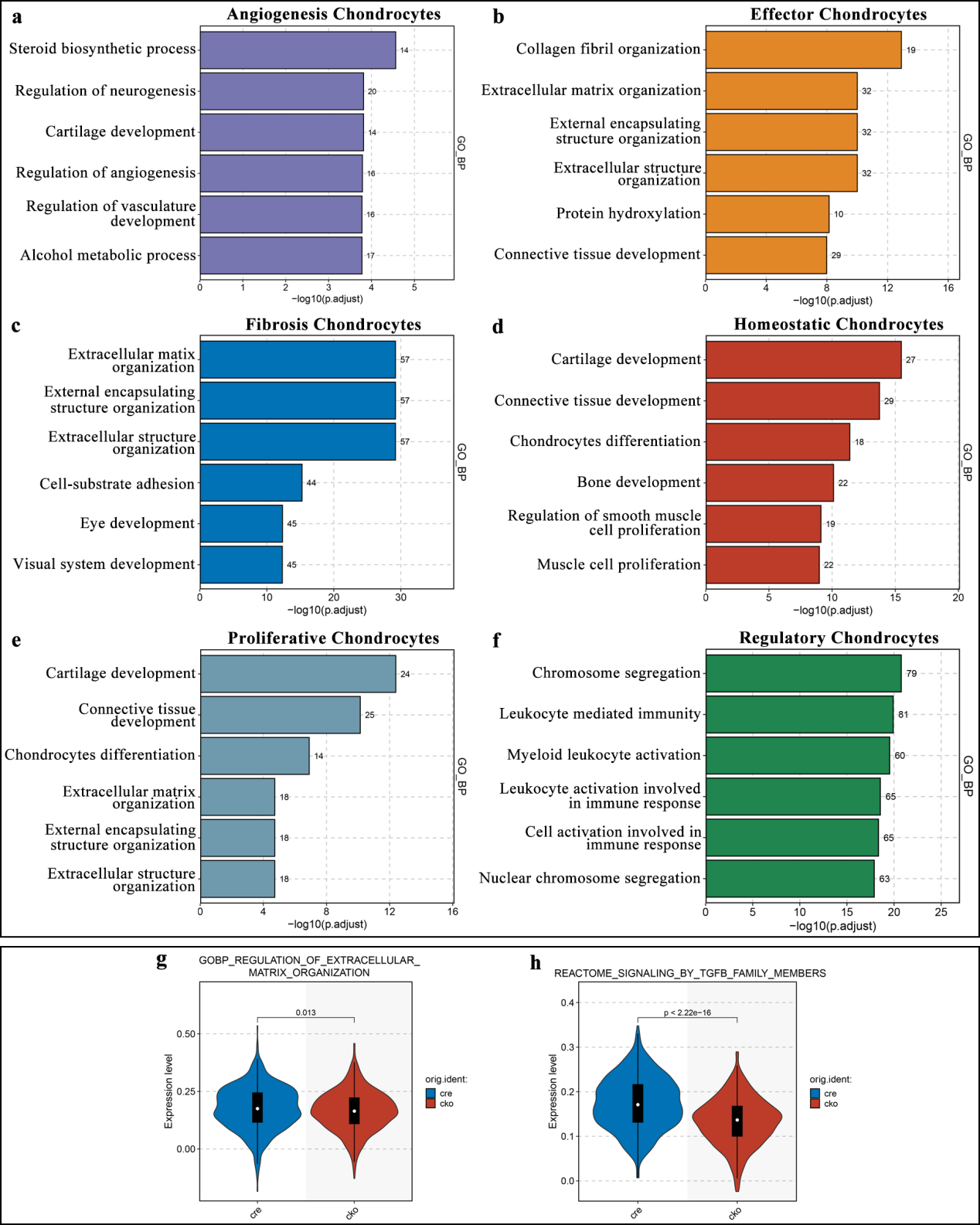
**

**Supplement-Figure-2: a-f.** GO enrichment analysis of the six chondrocyte subpopulations identified by single-cell RNA sequencing. The top enriched GO terms are ranked by statistical significance, with numbers indicating the count of enriched genes: (a) Angiogenesis Chondrocytes, (b) Effector Chondrocytes, (c) Fibrosis Chondrocytes, (d) Homeostatic Chondrocytes, (e) Proliferative Chondrocytes, and (f) Regulatory Chondrocytes. **g, h.** Violin plots showing the distribution of bioinformatic fibrosis scores based on pathway signature expression across all chondrocytes in Cre and cKO groups: (g) Regulation of extracellular matrix organization and (h) TGF-β family signaling.

**Supplement-Figure-3**


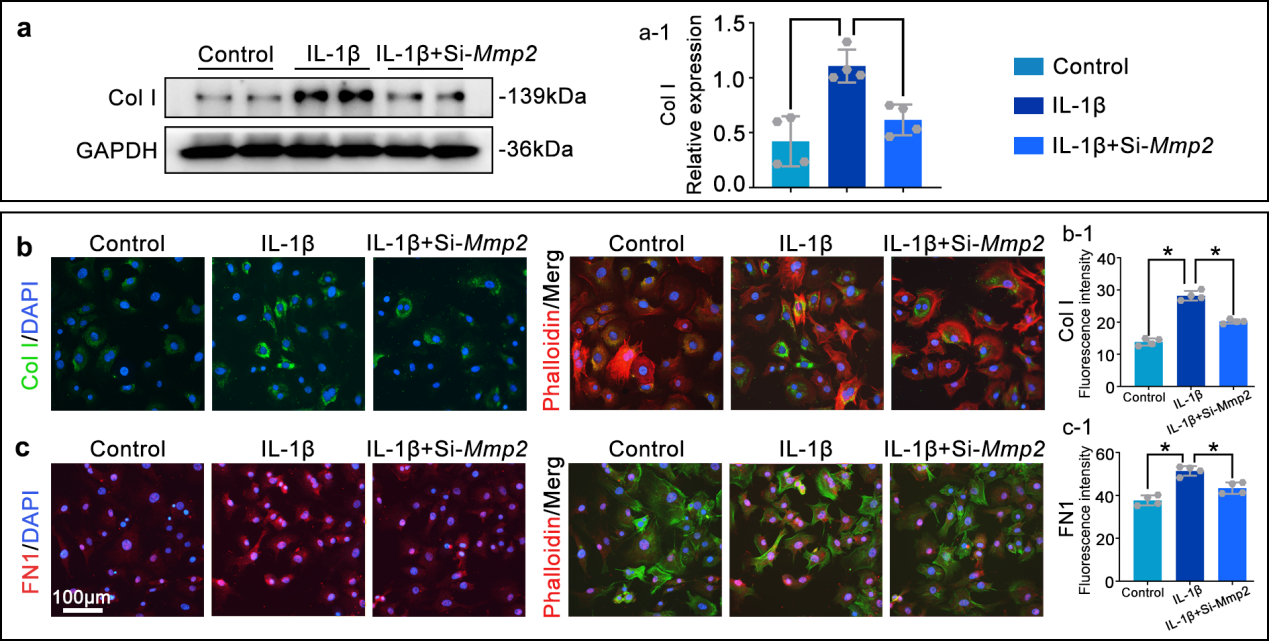


**a.** Western blot analysis of Collagen I expression in primary chondrocytes treated with control medium, IL-1β, or IL-1β+Si-*Mmp2*. **b, c.** Immunofluorescence analysis of primary chondrocytes under indicated treatments: **b.** Collagen I (green) and phalloidin-labeled F-actin (red). **c.** Fibronectin 1 (red) and phalloidin-labeled F-actin (green).

**Supplement-Figure-4**
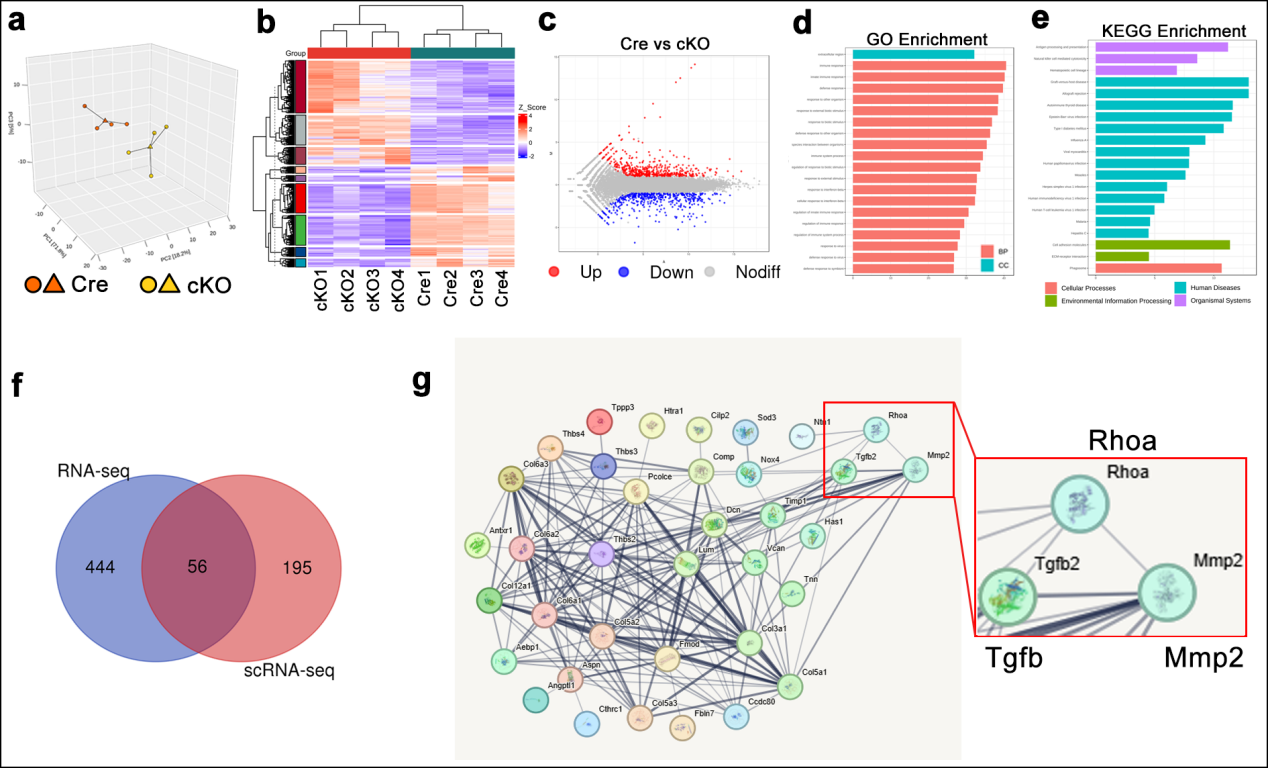


**Supplement-Figure-4: a-e.** Transcriptome sequencing of articular cartilage tissue from OA joints (Cre and cKO groups), including PCA, heat map, volcano plot, GO enrichment, and KEGG functional enrichment. **f.** Cross-analysis of transcriptomic results of cartilage tissue from Cre and cKO groups with single-cell sequencing results, identifying 56 intersecting genes. **g.** STRING database prediction of protein-protein interactions for the 56 intersecting genes.

**Supplement-Figure-5**


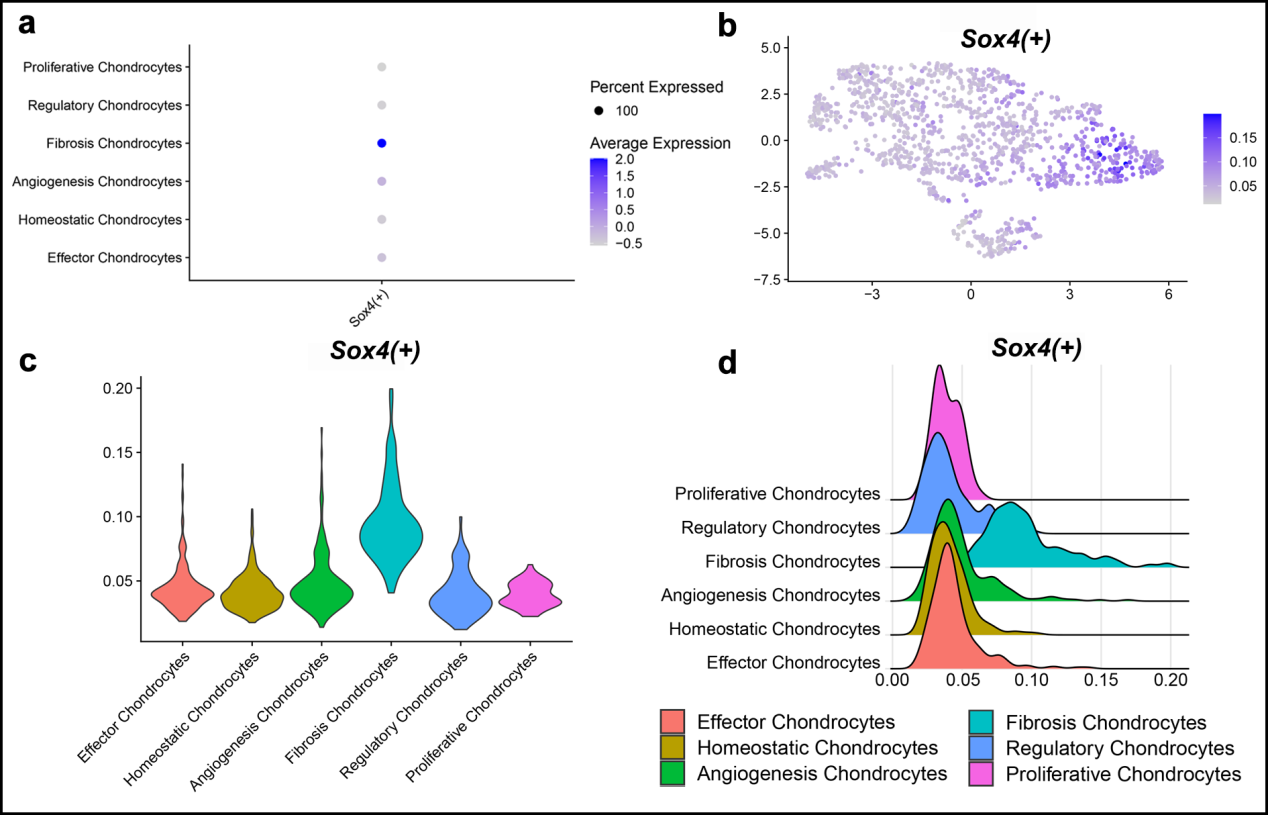


**Supplement-Figure-5: a-d.** Single-cell sequencing analysis showing that *Sox4* effectively targets the fibrocartilage chondrocytes population.
