# Supplementary material for: RhoA deficiency in chondrocyte inhibits cartilage fibrosis and ameliorates osteoarthritis progression via SOX4/MMP2 axis
Source: J Orthop Translat. 2026 May 14;58:101127. doi: 10.1016/j.jot.2026.101127 (PMC13206727; doi:10.1016/j.jot.2026.101127)
Supplement: Multimedia component 3 [file mmc3.docx]

**Supplement-Table-1**

| **Antibody** | **Source species** | **Dilution** | **Company** |
| --- | --- | --- | --- |
| β-Actin | Rabbit | 1:1000 (WB) | Abcam, UK, Cat#abab8226 |
| Collagen I | Rabbit | 1:1000(WB),1:100(IF) | Proteintech, USA, Cat#14695-1-AP |
| Collagen II | Mouse | 1:1000(WB),1:100(IF) | Proteintech, USA, Cat#12789-1-AP |
| Fibronectin 1 | Rabbit | 1:1000(WB),1:100(IF) | Zenbio, CHN, Cat#250073 |
| GAPDH | Rabbit | 1:1000 (WB) | Abcam, UK, Cat# ab8245 |
| mDia | Rabbit | 1:1000 (WB) | Zenbio, CHN, Cat#R383195 |
| MLCK | Rabbit | 1:1000 (WB) | Abcepta, USA,Cat#AP7966A |
| MMP2 | Rabbit | 1:1000(WB),1:100(IF) | Proteintech, USA, Cat#10373-2-AP |
| RhoA | Rabbit | 1:1000(WB),1:100(IF) | Santa, USA, Cat#sc-418 |
| ROCK | Mouse | 1:1000(WB) | Abcam, UK, Cat#ab125025 |
| SOX4 | Mouse | 1:1000(WB),1:100(IF) | Bioss, CHN, Cat#bs-11208R |
| α-SMA | Rabbit | 1:1000 (WB) | Abclonal, CHN, Cat#A2235 |
| TGF-β | Rabbit | 1:1000 (WB) | Abcam, UK, Cat#ab124894 |
| HRP-conjugated secondary antibody | Rabbit | 1:2000 (WB) | Molecular Probes, USA, Cat#65-6120 |
| HRP-conjugated secondary antibody | Mouse | 1:2000 (WB) | Invitrogen, USA,  Cat#SA5-10317 |
| Goat anti-rabbit Alexa Fluor 568 | Goat | 1:400 (IF) | Invitrogen, USA,  Cat#A-11011 |
| Goat anti-mouse Alexa Fluor 488 | Goat | 1:400 (IF) | Invitrogen, USA,  Cat#A-21121 |

**Supplement-Table-1.** The list of antibodies used in present study.

Note: **WB** means western blot, **IF** means immunofluorescence.
